# Genome-wide SNP data reveal hidden hierarchical population structure and demographic history of endangered black-and-white snub-nosed monkeys (*Rhinopithecus bieti*)

**DOI:** 10.1093/molbev/msag104

**Published:** 2026-04-17

**Authors:** Yitian Li, Zhiru Xu, Yingli Jiang, Minglin Chen, Yuan Li, Fan Liu, Jia Luo, Jiachao Feng, Weimin Kuang, Li Yu

**Affiliations:** School of Life Sciences, State Key Laboratory for Conservation and Utilization of Bio-Resource in Yunnan, Yunnan University, Kunming 650500, China; School of Life Sciences, State Key Laboratory for Conservation and Utilization of Bio-Resource in Yunnan, Yunnan University, Kunming 650500, China; School of Life Sciences, State Key Laboratory for Conservation and Utilization of Bio-Resource in Yunnan, Yunnan University, Kunming 650500, China; School of Life Sciences, State Key Laboratory for Conservation and Utilization of Bio-Resource in Yunnan, Yunnan University, Kunming 650500, China; School of Life Sciences, State Key Laboratory for Conservation and Utilization of Bio-Resource in Yunnan, Yunnan University, Kunming 650500, China; School of Life Sciences, State Key Laboratory for Conservation and Utilization of Bio-Resource in Yunnan, Yunnan University, Kunming 650500, China; School of Life Sciences, State Key Laboratory for Conservation and Utilization of Bio-Resource in Yunnan, Yunnan University, Kunming 650500, China; School of Life Sciences, State Key Laboratory for Conservation and Utilization of Bio-Resource in Yunnan, Yunnan University, Kunming 650500, China; School of Life Sciences, State Key Laboratory for Conservation and Utilization of Bio-Resource in Yunnan, Yunnan University, Kunming 650500, China; School of Life Sciences, State Key Laboratory for Conservation and Utilization of Bio-Resource in Yunnan, Yunnan University, Kunming 650500, China; Southwest United Graduate School, Kunming 650500, China

**Keywords:** *Rhinopithecus bieti*, fecal DNA, population structure, genetic diversity, landscape connectivity, demographic history

## Abstract

The endangered black-and-white snub-nosed monkey (*Rhinopithecus bieti*), endemic to high-altitude forests in southwest China, has increased from fewer than 1,500 individuals pre-1990 to over 3,500 post-2010. However, it faces severe habitat fragmentation, with at least 20 isolated groups. A comprehensive investigation of large-scale population genomic analysis is lacking. We present the first comprehensive genomic reassessment of this species using fecal-DNA and targeted capture sequencing to generate genome-wide single-nucleotide polymorphism data for 309 individuals. We identified five distinct genetic populations (Southwest, Southeast, Central, North-Central, and North) with strong geographic associations. Furthermore, we found previously unrecognized subpopulations, primarily associated with isolation within human-altered landscapes. Roads and human settlements were the primary barriers to contemporary genetic connectivity. Genetic diversity is highest centrally and declines peripherally, reflecting historical/recent barriers. Demographic inference suggests: (i) a possible southwestern origin and northward dispersal at ∼128.5 to 8.2 ka, probably driven by late Pleistocene climatic oscillations and local refugia; (ii) major subpopulation divergences within the last ∼610 to 120 years ago, likely due to human exploitation; and (iii) a sharp decline ∼300 years ago, leaving extremely low effective population size (40 to 314). Admixture origins of both Southeast and North-Central populations highlight their role in facilitating gene flow. Historically, habitats with high connectivity contrast with current severe fragmentation, particularly in the southern regions; this persistent suitability disparity suggests limited historical connectivity promoting genetic divergence between southern and central/northern populations. Our results provide critical insights into the population structure and evolutionary history of *R. bieti*, offering critical insights for conservation and demonstrating the power of fecal genomics in endangered species.

## Introduction

**Figure 1:**
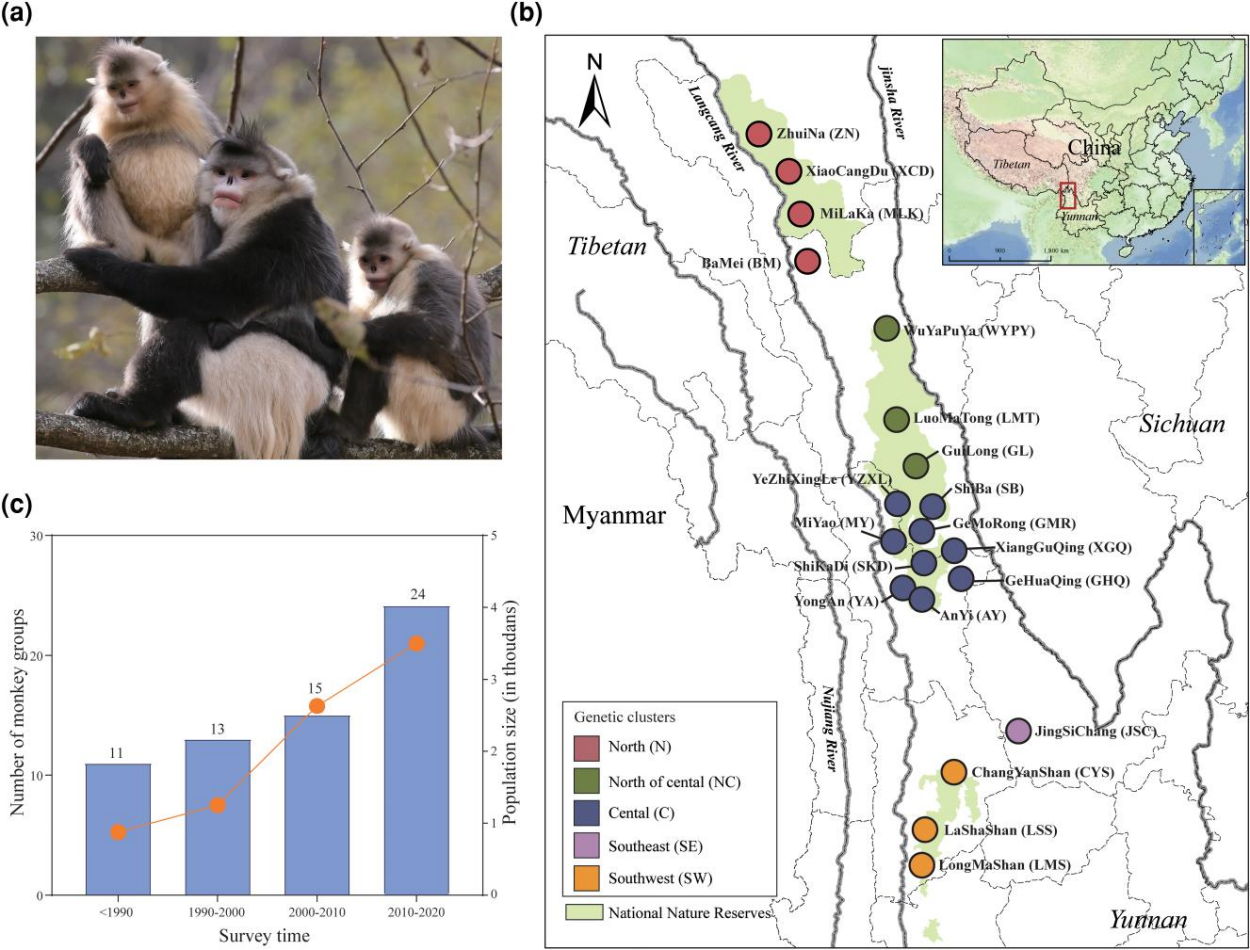
Map of sampling locations and population survey for *R. bieti*. a) *R. bieti*, photographed by Xinming He. b) Sampling locations. Populations correspond to the genetic structure shown in Fig. 2. Groups were categorized into five distinct genetic clusters: Northern (N; red), North of central (NC; dark green), Central (C; blue), Southeastern (SE; purple), and Southwestern (SW; orange). c) Population survey across four survey periods (<1990; 1990 to 2000; 2000 to 2010; 2010 to 2020). The bars represent the number of monkey groups per period, while the line denotes the census population size.

The black-and-white snub-nosed monkey (*Rhinopithecus bieti*), endemic to a narrow elevational corridor between the Lancang (Mekong) and Jinsha (upper Yangtze) rivers along the eastern Tibetan Plateau edge (Li et al. 1981; Yang 1988; Long et al. 1994; Xiao et al. 2003; Grueter et al. 2008; Huang et al. 2017), still remains endangered despite a significant population increase—from approximately 1,000 to 1,500 individuals across 11 monkey groups to over 3,500 in more than 20 groups (Long et al. 1994; Su et al. 2022; Li and Kuang 2026) (Fig. 1). The species is experiencing severe habitat loss and fragmentation crises caused by human activities (e.g. road construction, deforestation, and agricultural expansion) (Xiao et al. 2003; Huang et al. 2008; Quan et al. 2011; Zhu et al. 2022), confining monkey groups to isolated patches and reducing genetic connectivity (Liu et al. 2007, 2009 ), making the species more vulnerable to extinction.

Investigating spatial patterns of population structure, genetic diversity, and demographic history provides crucial insights into the genetic viability and conservation status of species in fragmented habitats, facilitating the planning of conservation and management strategies (Frankham 2005; Funk et al. 2012; Buckland et al. 2014; Hohenlohe et al. 2021; Yang et al. 2022). Previous population genetic studies on *R. bieti* have been constrained by (i) restricted sampling coverage (e.g. inclusion of only 11 groups), failing to incorporate newly documented populations; and (ii) reliance on low-resolution molecular markers (such as mitochondrial DNA [mtDNA] and microsatellites) that yielded inconsistent assessments of population structure (Liu et al. 2007, 2009, 2015). mtDNA analyses identified two major lineages (Liu et al. 2007), including a widespread northern/central/southwestern clade (Haplogroup A) and a restricted southeastern clade (Haplogroup B), while microsatellite data suggested five distinct genetic clusters (Northwest, Northeast, Central, Southeast, and Southwest clades) (Liu et al. 2009). Furthermore, knowledge of demographic history remained limited due to these marker limitations, preventing resolution of recent historical fluctuations, population divergence, and gene flow dynamics. These constraints have prevented the formulation of a contemporary, range-wide understanding of the species’ genetic architecture.

Here, we present the first comprehensive genomic reassessment of the endangered *R. bieti* using targeted genome-wide single-nucleotide polymorphism (SNP) genotyping data (51,182 SNPs) across 309 individuals from 20 known monkey groups, including recently discovered monkey groups. This approach enables: (i) resolution of contemporary patterns of population structure and genetic diversity across the species’ entire range; (ii) assessment of migration rates across the landscape heterogeneity to detect potential barriers to gene flow; (iii) detailed modeling of historic dispersal and reconstruction of recent demographic dynamics, and determining habitat suitability for *R. bieti*. Our results provide unprecedented genomic insights and essential information to guide effective conservation and management strategies for this species.

## Results

### Sampling and SNP calling

A total of 494 fecal samples were collected from 20 locations across the distribution range of *R. bieti* (Fig. 1). We employed targeted capture sequencing (TargetSeq), a cost-effective next-generation sequencing method for low-quality fecal DNA (Fontsere et al. 2021, 2022; Li et al. 2025), to enrich target DNA and detect genetic variation. First, 54,892 putatively neutral autosomal regions were selected from across the genome, and corresponding probes were designed. After hybridization capture and high-throughput sequencing of all fecal samples, mean coverage depth across target regions was 22.3-fold (range: 2.0- to 279.5-fold), with an average of 84.9% (range: 20.2% to 99.9%) target positions covered per sample (Table S1). Following quality control, we excluded 164 low-quality samples and 21 samples identified as first-degree relatives. The final neutral dataset comprised 309 samples genotyped at 51,182 nuclear biallelic SNPs (mean depth = 28.84-fold, mean coverage = 94.95%), which were used for subsequent population genomic analysis.

### Phylogenetic relationships and population structure

**Figure 2:**
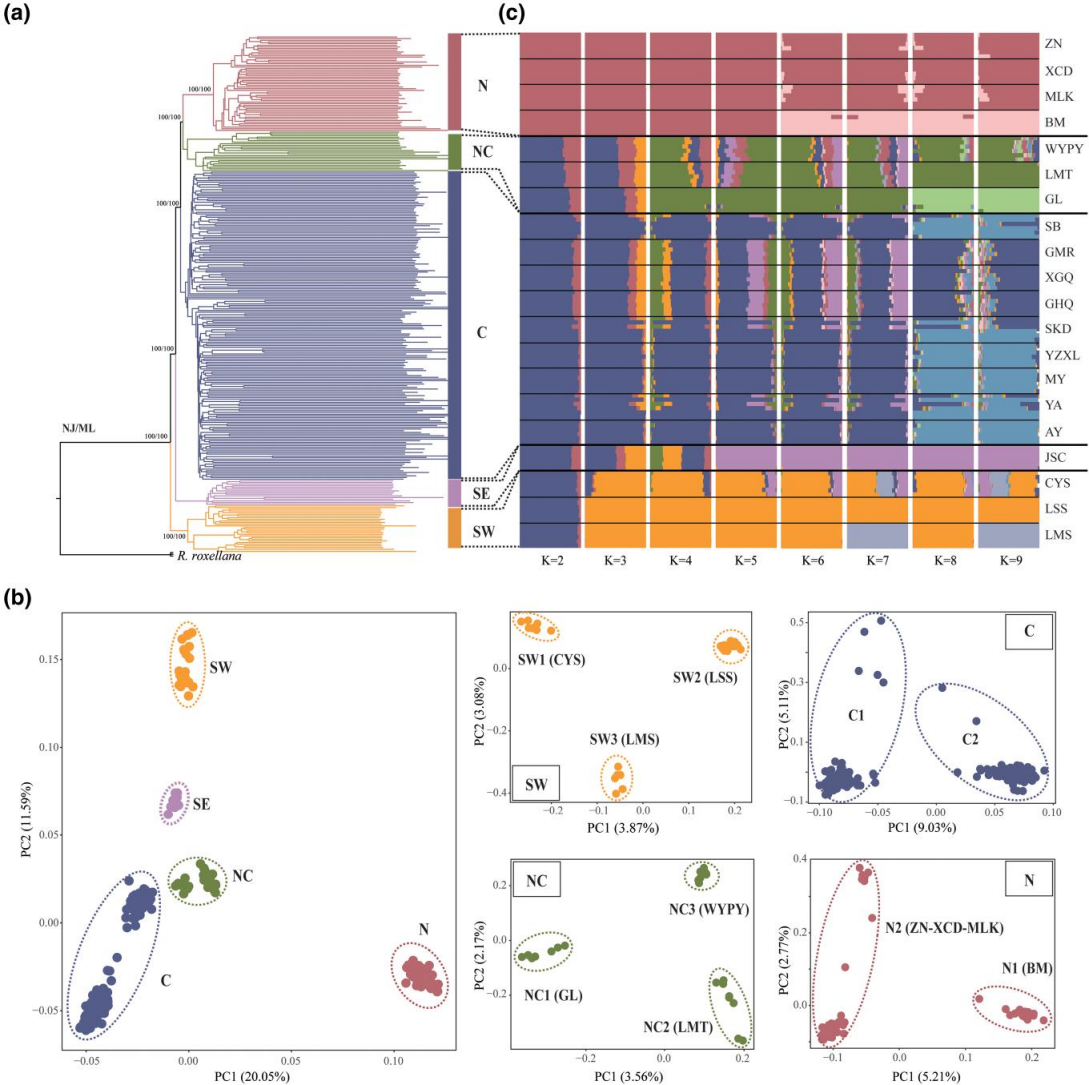
Range-wide population genomic structure of *R. bieti*. a) Individual-based neighbor-joining (NJ) and maximum likelihood (ML) reconstructions. NJ and ML bootstrap values are given at branches (values < 80% are not shown). b) PCA plot for the first two principal components (PC1 and PC2) showing the genetic structure and substructure based on all individuals and independent PCAs for each cluster. Each point represents one individual. c) Population genetic structure inferred in ADMIXTURE for *K* = 2 to 9 with color representing ancestry components. The black line separates samples from 20 monkey groups.

We examined patterns of population clustering across the whole habitat range of *R. bieti* by phylogenetic reconstruction, principal component analysis (PCA), and ADMIXTURE analysis (Fig. 2). Both individual-based neighbor-joining (NJ) and maximum-likelihood (ML) phylogenetic analysis consistently identified five main genetic clusters (Fig. 2a), corresponding to samples from the southwestern (SW: CYS, LSS, and LMS groups), southeastern (SE: JSC group), central (C: SB, GMR, XGQ, GHQ, SKD, YZXL, MY, YA, and AY groups), north-central (NC: WYPY, LMT, and GL groups), and northern (N: ZN, XCD, MLK, and BM groups) ranges, respectively. These clusters align with distinct landscape patches and are separated by natural and/or anthropogenic barriers. All five genetic clusters and their phylogenetic relationships received 100% bootstrap support. In addition, individual-based phylogenetic analysis placed all individuals from SW cluster in a separate clade, highlighting their marked genetic distance from the remaining *R. bieti* populations. The SW, NC, and N clusters tended to form subpopulations corresponding to sampling locations. In contrast, both SE and C genetic clusters tended to form their own clade, with more than 90% support, little phylogenetic structure according to sampling groups, and also including a few N populations (ZN, XCD, and MLK groups). Both individual-based and population-based phylogenetic relationships based on TreeMix method suggested gradual northward divergence patterns, with SW cluster diverging first from the other populations, followed by SE cluster, and subsequently C cluster diverging before NC and N clusters (Fig. 2a and Figure S1).

PCA analysis based on 309 *R. bieti* individuals further supported the existence of five main genetic clusters: SW, SE, C, NC, and N. The first and second principal components accounted for 20.05% and 11.59% of the total genetic variation, respectively, with samples clustering broadly according to their geographic proximity (Fig. 2b). To eliminate the clustering bias caused by different sample sizes, we performed random downsampling to generate equal-sized subsets with varying gradients from each cluster (Figure S2). Subsequently, independent PCAs focusing on SW, C, NC, and N clusters not only confirmed this overall pattern but also revealed finer-scale substructures, as follows. The SW cluster comprised three subpopulations (SW1: CYS; SW2: LSS; SW3: LMS). The C cluster consisted primarily of two subpopulations (C1: SB, GMR, XGQ, GHQ, SKD, and YA; C2: SB, YZXL, MY, SKD, YA, and AY), which roughly correspond to eastern and western geographic groups, although they showed no genetic clustering according to sampling location. The NC cluster contained three potential subpopulations (NC1: GL; NC2: LMT; NC3: WYPY). The N cluster generally included two subpopulations (N1: BM; N2: ZN, XCD, and MLK). These results demonstrate the ability of large sample sizes based on whole-genome SNP data to explore fine-scale population structures.

In model-based clustering analysis using ADMIXTURE (Alexander et al. 2009) based on subsamples of the same size, the cross-validation (CV) error across 200 independent runs showed that *K* = 4 had the lowest values, supporting four genetic clusters (N, NC, C/SE, and SW; Fig. 2c and Figure S3). Notably, this ADMIXTURE grouping differed from those identified by PCA and phylogenetic reconstruction analysis, as it merged SE and C clusters into a single genetic unit. This discordance probably reflects substantial genetic admixing between SE and both C and SW clusters. Although *K* = 4 yielded the lowest CV value, similar values were also found at *K* = 3 and 5 (Figure S3). At *K* = 3, samples were divided into three clusters: (i) NC, C, and SE formed a cluster, with pronounced genetic admixing, with (ii) SW, and (iii) N forming their own genetic clusters, respectively. At *K* = 5, the ADMIXTURE clustering pattern largely agreed with PCA and phylogenetic results. In addition, we followed the recommendations of previous authors using ADMIXTURE analysis (Alexander and Lange 2011; Frichot and Francois 2015; Tumendemberel et al. 2023), in order to obtain biologically meaningful information about relationships among groups, and also evaluated higher *K* values (*K* > 5). At *K* = 6, BM group diverged from N cluster, and at *K* = 7, LMS group split from SW cluster. As *K* values increased, further substructure emerged: GL group split from NC cluster, and within SW cluster, CYS and LSS groups separated. Overall, these results revealed fine-scale subpopulations, consistent with independent PCA results (Fig. 2b).

### Genetic differentiation, gene flow, and landscape connectivity

**Figure 3:**
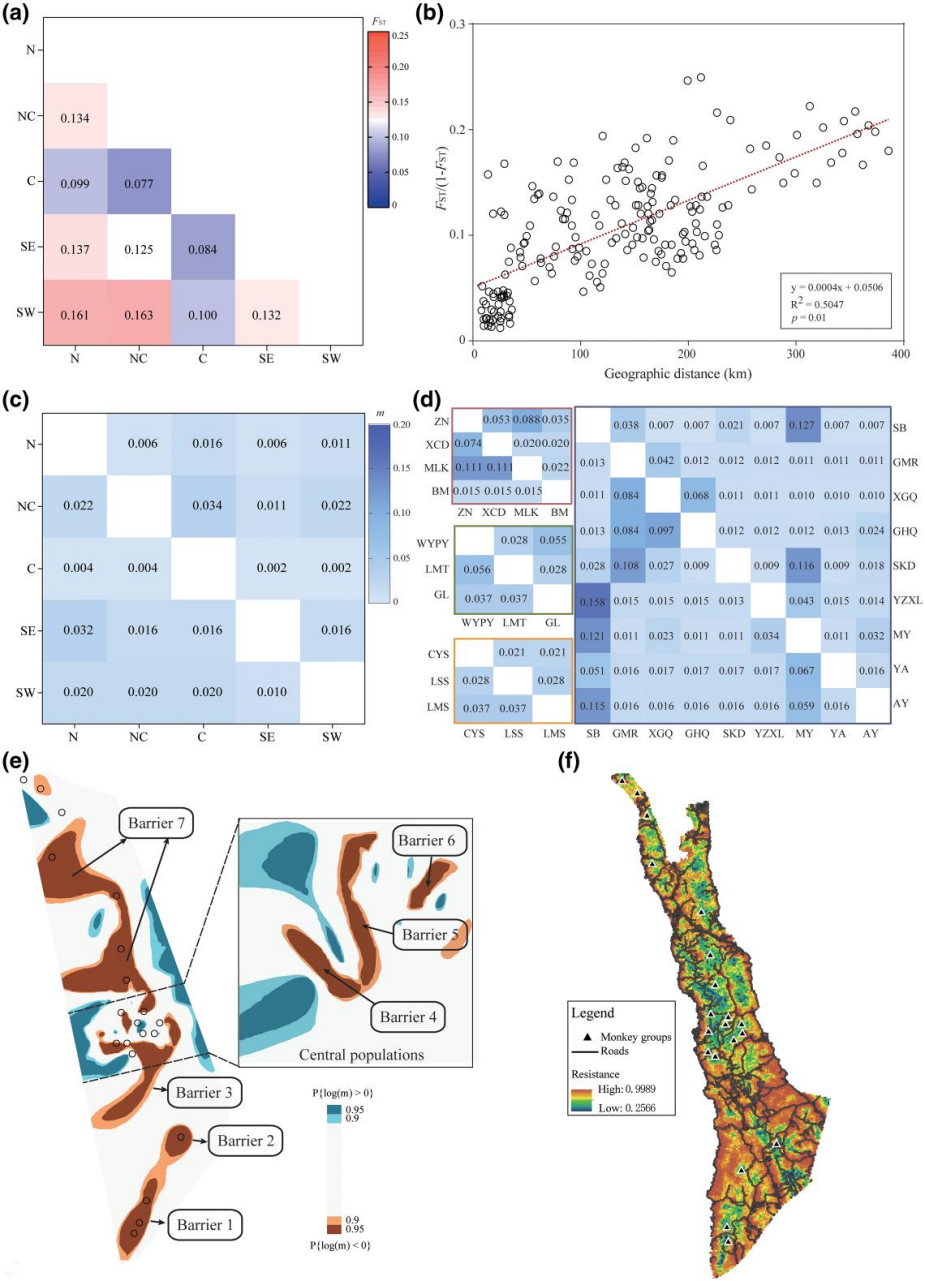
Genetic differentiation and gene flow among subpopulations of *R. bieti*. a) Pairwise genetic differentiation (*F*_ST_) between the five genetic clusters (N, NC, C, SE, and SW). Red indicates high genetic diversity, while blue indicates low genetic diversity. b) Comparisons of standardized *F*_ST_ values with geographic distance between locations to test for isolation by distance (IBD) across the species’ range. Euclidean geographic distance explains 50.47% of the observed genetic variation (*R*^2^ = 0.505, *P* = 0.01). c) Pairwise estimated contemporary migration rates (*m*) among five genetic clusters, and d) among monkey groups using the BA3-SNPs method. e) Effective migration surfaces and main barriers inferred by EEMS, and independent EEMS analysis in the central population, with a threshold of 0.9. Blue values indicate regions of relatively high effective migration, while orange values indicate regions of relatively low effective migration. f) Landscape resistance surfaces inferred by MaxEnt. Orange indicates areas of high resistance, while green represents areas of low resistance. Black triangles denote sample locations. The road networks are shown in black lines based on data from www.openstreetmap.org.

The pairwise genetic differentiation (*F*_ST_) estimates further supported the population genetic clusters identified by phylogenetic, PCA, and ADMIXTURE analysis, revealing moderate to high levels of genetic differentiation between groups within five major clusters (*F*_ST_ = 0.077 to 0.163; Fig. 3a). In contrast to the moderate genetic differentiation observed between C and other clusters (*F*_ST_ = 0.077 to 0.100), the remaining clusters exhibited high genetic differentiation (*F*_ST_ = 0.125 to 0.163). Within each cluster, genetic differentiation was highest among the SW locations (*F*_ST_ = 0.106 to 0.135) and among NC locations (*F*_ST_ = 0.083 to 0.142), whereas C localities exhibited minimal differentiation (*F*_ST_ = 0.012 to 0.048) (Figure S4). For all sampling sites included in the isolation by distance (IBD) analysis, a Mantel test showed a strong and statistically significant correlation between genetic distance and geographic distance (*R*^2^ = 0.505, *P* = 0.01; Fig. 3b).

We evaluated recent migration rates (*m*) using a Bayesian framework implemented in BA3-SNPs (Wilson and Rannala 2003; Mussmann et al. 2019). This analysis suggested that there is no significant contemporary gene flow between five clusters, with estimated *m* ranging from 0.001 to 0.034 (Fig. 3c), while there are moderate contemporary migration rates within clusters overall (*m* = 0.011 to 0.158; Fig. 3d). Results from Estimated Effective Migration Surfaces (EEMS) (Petkova et al. 2016) further revealed moderately low average effective migration across the species’ range, showing a significant signal of genetic discontinuity and pronounced spatial genetic structure (Fig. 3e). At least seven effective local barriers to gene flow (barriers 1 to 7; Fig. 3e) were identified both between and within the five genetic clusters, most of which coincided with human-modified landscapes (Fig. 3f). Results of landscape resistance analysis showed that human-related variables collectively account for 65.9% of the variation in connectivity, with human settlements (30.7%), human population density (22%), and roads (10.1%) being the strongest contributors (Table S2). The resulting resistance pattern is closely aligned with the migration surface inferred from EEMS, particularly showing higher resistance in the southern ranges. To further evaluate how specific landscape features affect gene flow, we performed landscape genetic analysis using ResistanceGA (Peterman 2018). Univariate analysis identified roads as the strongest single factor affecting genetic connectivity (AIC = 240.63; Table S3). Multivariate model analysis, which included all possible variable combinations and a null Euclidean model, showed that the model containing roads and human settlements provided the best fit (AIC = 242.65; Table S4). Overall, our findings indicated that human activities, especially roads and human settlements, were the principal drivers of contemporary genetic discontinuities in *R. bieti* populations across its distribution range.

### Genetic diversity

**Figure 4:**
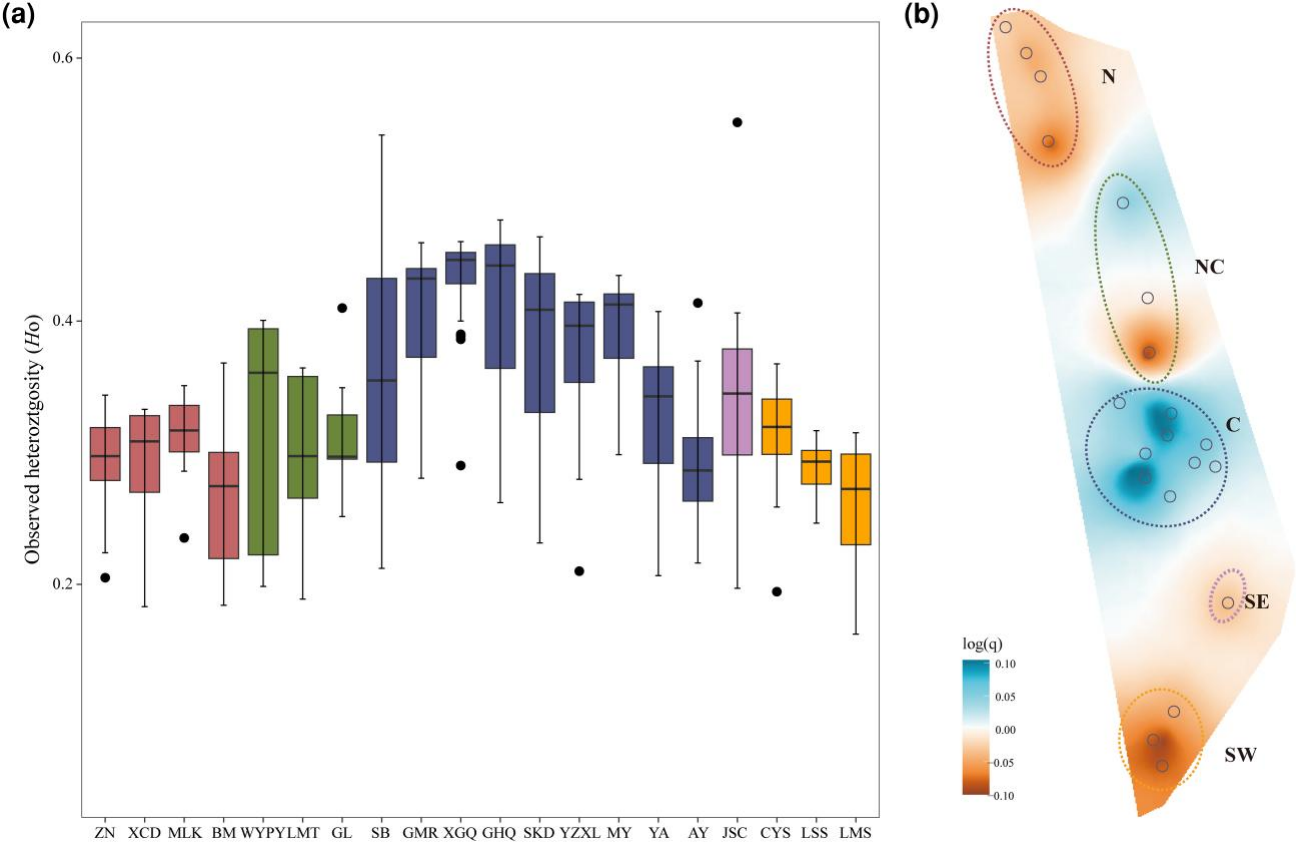
Genetic diversity of *R. bieti*. a) Genetic diversity estimated as the proportion of observed heterozygosity (*H*o) per individual. b) Spatial distribution of genetic diversity inferred by EEMS.

Average observed autosomal heterozygosity (*H*o) across sampled locations was moderate (*H*o = 0.347). In comparison, the C population exhibited the highest level of genetic diversity (*H*o = 0.379), followed by NC (*H*o = 0.342) and SE (*H*o = 0.312), whereas peripheral SW (*H*o = 0.291) and N (*H*o = 0.287) populations showed substantially lower diversity (Fig. 4a and Figure S5). Within the C cluster, we also noted considerable variation in heterozygosity: core groups exhibited higher diversity, while groups in locally restricted or barrier-affected habitats had relatively lower genetic diversity (Fig. 3d). Notably, SB and SKD groups from the C cluster displayed marked heterogeneity in heterozygosity, suggesting its genetic composition likely derives from two distinct subpopulations (Fig. 2b), possibly as a result of continuous migration from neighboring groups. Spatial patterns of genetic diversity were further supported by EEMS analysis (Fig. 4b). Finally, we examined the relationship between *H*o and census population size across monkey groups, but found no significant correlation (*P* = 0.126; Figure S6).

### Recent demographic history

**Figure 5:**
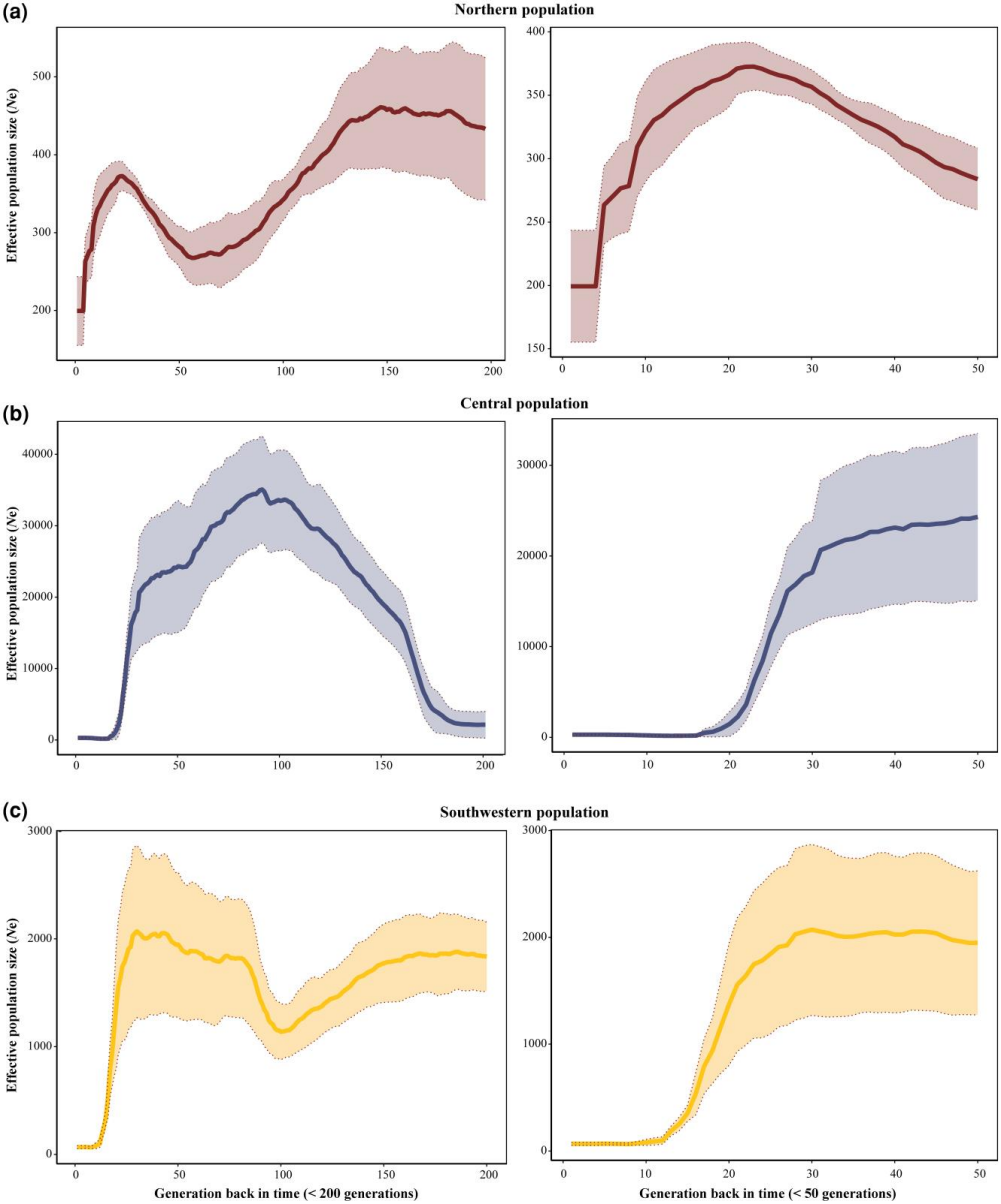
Recent demographics of the Northern (a), Central (b), and Southwestern (c) populations for the past 200 and 50 generations using GONE (Santiago et al. 2020). The lines represent median *N*_e_ estimates, while the shaded areas represent the 95% confidence intervals.

Inference of recent changes in *N*_e_ using linkage disequilibrium (LD) patterns across the genomes revealed population fluctuations in the N, C, and SW clusters (Fig. 5). We restricted our analysis to the past 200 generations, as the GONE (Santiago et al. 2020) method demonstrates optimal reliability within this timeframe. The N cluster experienced a gradual decline in *N*_e_ starting ∼130 generations ago, reaching its lowest point between ∼70 and ∼50 generations ago and subsequently increasing until ∼20 generations ago, after which it declined again (Fig. 5a). In the C cluster, *N*_e_ gradually increased from ∼170 to ∼90 generations ago, followed by a gradual decline (Fig. 5b). The SW cluster gradually increased between ∼150 and ∼100 generations ago, remained relatively stable from ∼70 to ∼20 generations ago, and then declined (Fig. 5c).

### Model comparison of migration events inference

We used a composite-likelihood approach implemented in Fastsimcoal2 (Excoffier et al. 2013) to investigate the possible origins and migration history of *R. bieti* populations. Based on observed phylogenetic relationships and genetic diversity patterns, we first evaluated two ancestral population hypotheses: a possible SW origin (Figure S7a) or a possible C origin (Figure S7b) of *R. bieti*. Both demographic models represented founder-migration scenarios. The AIC results revealed that the possible SW origin was the more likely demographic scenario.

To further elucidate migration events among the five major clusters that we identified, we compared six demographic scenarios: stepping-stone dispersal model (Model 1; Figure S8a), stepping-stone dispersal with admixture origin of SE model (Model 2; Figure S8b), stepping-stone dispersal with admixture origin of NC model (Model 3; Figure S8c), stepping-stone dispersal with admixture origins of both SE and NC model (Model 4; Figure S8d), isolation-with-migration model (Model 5; Figure S8e), and independent migration model (Model 5; Figure S8f). AIC values revealed that the stepping-stone dispersal with admixture origins of both SE and NC populations provided the best-fitting demographic model for the data (AIC = 5,719,383), outperforming all alternative models (Model 1: AIC = 6,047,560; Model 2: AIC = 5,812,958; Model 3: AIC = 5,812,131; Model 5: AIC = 6,036,734; Model 6: AIC = 5,793,425; Table S5). We further validated the best-fitting model by comparing the expected and observed 2D- and 1D-SFSs, and matching the shapes of the SFSs and the levels of genetic variation (Figure S9).

**Figure 6:**
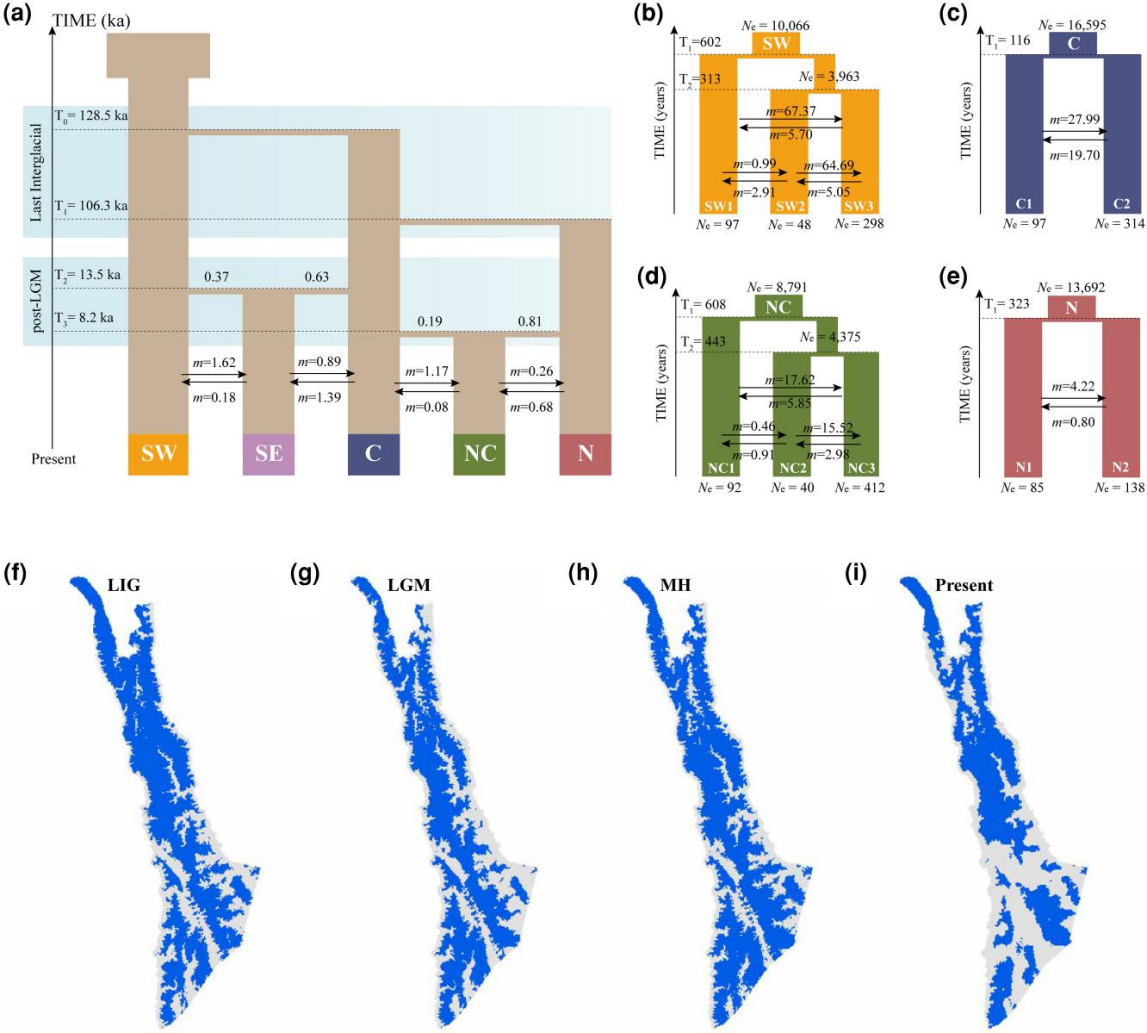
Demographic models and ecological niche models of *R. bieti*. a) Demographic scenario of five *R. bieti* populations inferred from the demographic model simulations, with estimates of divergence time (T) and migration rates (*m*, number of migrants per generation). Divergence simulation within the SW (b), C (c), NC (d), and N (e) clusters. Results of the modeling past and present range dynamics, including the last interglacial (LIG, 129 to 116 ka; f), the last glacial maximum (LGM, 27 to 19 ka; g), the middle Holocene (MH, ∼8.2 to 4.2 ka; h), and the present (1970 to 2000 AD; i). Areas in blue indicate high habitat suitability (index values > 0.1), and grey areas represent less suitable habitats for this species. Bioclimatic variables included in the models: mean diurnal range (BIO2), isothermality (BIO3), max temperature of warmest month (BIO5), and temperature annual range (BIO7).

For the best-fitting demographic model, the estimated divergence times mainly fell within the last interglacial (LIG; ∼128.5 to 106.3 ka) and the warm period following the end of the last glacial maximum (LGM; ∼13.5 to 8.2 ka; Fig. 6a). The specific inferences are as follows: (i) The C population first diverged from the SW population at ∼128.5 ka (95% confidence interval [CI]: ∼107.7 to 139.3 ka); (ii) Subsequently, the C population diverged from the N population at ∼106.3 ka (95% CI: ∼95.2 to 118.6 ka); (iii) The admixed origins of the NC and SE populations were formed at ∼13.5 ka (95% CI: ∼11.2 to 15.4 ka) and ∼8.2 ka (95% CI: ∼6.5 to 10.8 ka), respectively. The SE population originated from the admixture of ∼37% SW and ∼63% C populations, and the NC population originated from the admixture of ∼19% C and ∼81% N populations. This result suggests that the admixed origins within the SE and NC populations may have served as key genetic stepping stones, playing an important role in gene flow among populations.

Furthermore, we conducted detailed demographic simulations to model divergence and gene flow within the four genetic clusters (SW, C, NC, and N; Fig. 5b to e). These analyses reveal that the divergences between subpopulations within each cluster are relatively recent (occurring ∼610 to 120 years ago). Furthermore, the models indicate that moderate to high levels of gene flow (0.46 to 67.37 migrants per generation) have been maintained following these splits. Estimates of *N*_e_ were consistently low across all subpopulations (*N*_e_ = 40 to 314).

### Reconstruction of past range dynamics

We employed ecological niche models (ENM; Phillips et al. 2006) to reconstruct habitat suitability for *R. bieti* during past climatic periods: the LIG (∼129 to 116 ka), LGM (∼27 to 19 ka), middle Holocene (MH; ∼6 ka), and the present (1970 to 2000). Our ENM demonstrated high predictive accuracy (area under the receiver operating characteristic curve [AUC] = 0.77 to 0.82; Table S6). The final models incorporated four bioclimatic variables after the elimination of all correlated variables: mean diurnal range (BIO2; 31% contribution), max temperature of warmest month (BIO5; 30.3%), temperature annual range (BIO7; 7.1%), and isothermality (BIO3; 5.1%). Model projections indicated relatively stable habitat suitability with high historical connectivity during the LIG, LGM, and MH periods (Fig. 6f to h). In contrast, present habitats exhibit severe fragmentation, particularly in southern regions (Fig. 6i). Across all four periods, habitat suitability was consistently lower in southern areas than in the central/northern regions. This persistent disparity suggests limited historical habitat connectivity, which may have contributed to genetic divergence between southern populations (SW and SE) and those in central/northern regions.

## Discussion

In this study, we present the first whole habitat range and genome-wide analysis of the population structure and genetic diversity of *R. bieti*; this supports the existence of five main genetic populations, and includes previously unreported findings of fine-scale substructures. By broadening the landscape features and molecular scope of data collection, we can infer ecological contexts within and across regions that support connectivity or suggest hidden barriers to dispersal. We also draw upon these data to elucidate the historic dispersal and recent evolutionary history of *R. bieti* and consider the impacts of demographic history on contemporary genomic diversity. Finally, we discuss implications for the future management and conservation of this endangered primate species, which inhabits fragmented landscapes along the eastern Tibetan Plateau edges.

Our range-wide genomic analysis confirms five distinct genetic clusters (SW, SE, C, NC, and N) within *R. bieti* populations, which each completely isolated by natural and anthropogenic barriers. The lack of contemporary gene flow and the presence of strong dispersal barriers further support treating each cluster as a separate conservation unit. However, this pattern contrasts with mtDNA studies, which identified two haplogroups with no clear geographic structure. Such mitochondrial-nuclear discordance is likely the result of mitochondrial introgression and female-biased dispersal, which is consistent with our demographic simulations showing extensive historical gene flow through secondary contact (Fig. 6a). In addition, as predicted by geographic distance, our results demonstrated significant genetic differentiation between clusters, particularly between the southernmost populations and others. Although the C, NC, and N clusters are geographically proximate, they exhibit significant genetic divergence—a pattern consistent with regional isolation observed in various montane species of mammals, amphibians, reptiles, and birds in the Southwest Mountains and on the edges of the Tibetan Plateau (Hu et al. 2011; Yan et al. 2013; Li et al. 2015a, 2025; Chen et al. 2022; Yang et al. 2022; Jiao et al. 2024). These consistent cross-taxa findings suggest potential historical or contemporary dispersal barriers, probably driven by the unique topographic features of the Southwest Mountains or the edges of the Tibetan Plateau, cyclical glacial–interglacial periods, and fragmented habitats resulting from features created by increasing human activity.

Incorporating newly sampled groups from previously undocumented regions, including N (MLK), NC (LMT), C (GHQ, SKD, YZXL, MY, and YA), and SW (LSS), reveals that their genetic clustering corresponds closely with geographical distribution. Notably, the addition of the LMT group consolidates a distinct north-central clade consisting of WYPY, LMT, and GL, despite physical barriers such as the National Road 214 and human settlements (Liu et al. 2009; Li et al. 2015b; Zhu et al. 2022). This suggests historical gene flow and habitat connectivity, recently disrupted by anthropogenic fragmentation. Our genomic results contrast with earlier microsatellite-based clustering, which separated WYPY as a distinct group while associating GL with central populations (Liu et al. 2009). These findings refine conservation unit boundaries for both newly recorded and known populations, offering genomic evidence to support targeted conservation planning.

Interestingly, within the five genetic clusters, we detected previously unrecognized subpopulations, particularly in peripheral groups, although the SE cluster comprised only a single isolated group. In general, across the entire distribution range, our landscape genetic analyses demonstrated that human activities, particularly roads and human settlements, are the principal drivers of contemporary genetic structure in *R. bieti*. At a local scale, other human-induced disturbances and natural topographic features, as documented in earlier studies, may further contribute to group isolation. Within the N cluster, at least two hidden subpopulations were detected. The BM group is isolated by human settlements and the physical barrier of the Jiawu and Ningjing Snow Mountains (Xiao et al. 2005), likely limiting connectivity with other northern populations. Consistently, contemporary gene flow estimates revealed a weak genetic connection with other northern groups (migration rates < 0.05). This limited connectivity, coupled with reduced genetic diversity in BM group, suggests a significant historical bottleneck effect followed by genetic drift. A previous habitat assessment reported a 15.5% reduction in the extent of northern habitats from 1986 to 2006, concurrent with expansions in pasture (58.1%) and farmland (17.8%) (Yong et al. 2008), reflecting human-driven forest loss and habitat fragmentation in these regions (Xiao et al. 2005; Yong et al. 2008). Within the NC cluster, at least three subpopulations were detected, with relatively low migration rates and strong migration barriers between them (barrier 7; Fig. 2e). These subpopulations are endemic to the north part of the Yunnan Baima Snow Mountain Nature Reserve and are significantly geographic isolated, occupying fragmented habitats and resulting in high genetic differentiation. Historical records indicated the former presence of now-extinct monkey groups, such as the Dong’a group and those from the type specimen locality (Allen 1938; Li et al. 1981), suggesting that extensive hunting in the past may have caused local extinctions in this region. In addition, previous studies proposed that GL group was a recent derivative of LMT group (Dai et al. 2017). However, our population structure analysis revealed substantial divergence in their genetic composition, which does not support this hypothesis. The small census population size (fewer than 100 individuals) in GL group and the accelerating loss of genetic variation resulting from long-term genetic drift have probably led to genetic differentiation from neighboring groups. In the central areas of the *R. bieti* range, gene flow between populations is significantly higher than in other regions, suggesting the potential existence of migration corridors. However, EEMS analysis identified at least three potential migration barriers (barriers 4 to 6; Fig. 2e). Notably, the barrier 5, which aligns with the north–south-oriented Yunling Mountains, probably divides the C cluster into two subpopulations. This divergence is further intensified by surrounding dense human settlements (Zhu et al. 2022). In contrast, both SB and YZXL groups contain both C1 and C2 genetic components while demonstrating higher genetic diversity, suggesting a potential role as functional corridors connecting those subpopulations. Conversely, peripheral populations exhibit greater genetic differentiation due to stronger geographical isolation. As expected, we also detected three potential substructures within the southeastern population, the distribution of which aligns with the monkey groups sampled across the geographic regions. The absence of significant recent gene flow and the severe migration barriers between these substructures indicate their complete isolation in fragmented habitats. A previous study proposed connecting these isolated groups through a stepping-stone strategy (Wang et al. 2023); however, this strategy requires systematic and sustained tracking and monitoring in the future. Overall, our genome-wide SNP data with comprehensive sampling locations provide the power to detect finer-scale genetic substructures in *R. bieti* populations, and reveal that most barriers coincide with areas of human activity.

Our study refines the understanding of origins and dispersal of *R. bieti* populations. In contrast to prior mtDNA studies suggesting a possible central origin based on its higher haplotype diversity than other populations (Liu et al. 2007), our whole-genome analyses reveal a more complex demographic history. Phylogenetic reconstructions consistently position the SW population at the basal node across both individual- and population-level trees, with subsequent lineages diverging along a distinct south-to-north progression. This pattern strongly supports a possible southern origin followed by northward stepwise dispersal. Results from microsatellite markers analysis further confirm the earliest divergence of the SW population (Liu et al. 2009). Through coalescent simulations, the southern-origin model has a significantly better fit compared to the central-origin hypothesis. Crucially, while the C cluster exhibits elevated genetic diversity—previously interpreted as evidence of an origin center—our ADMIXTURE analyses (*K* = 3 to 5) and historical gene flow patterns indicate that this probably reflects secondary contact rather than ancestral status. Topological assessment provides definitive evidence: if the C cluster represents the ancestral population of *R. bieti*, individuals from here should dominate basal phylogenetic positions, an expectation that is not supported by our data. Thus, our findings do not support the central-origin hypothesis and establish a southern-origin paradigm. Furthermore, our demographic reconstruction of dispersal dynamics reveals a stepping-stone pattern from south to north, synchronized with climatic oscillations since the Late Pleistocene. Key divergence events include: the initial isolation of the C population ∼128.5 ka, followed by the genetic divergence between the N and C populations ∼106.3 ka. Both population expansion events correspond to interstadial periods within the last glacial period, suggesting that relatively warm climates drove population expansion into suitable habitats, while subsequent local refugia may have facilitated population divergence, mirroring dispersal patterns observed in avian species of the Southwest Mountainous regions (Dong et al. 2022). Furthermore, we further revealed that the SE and NC populations originated from admixture between adjacent populations, suggesting that they may have served as crucial “stepping-stone” populations, playing an important role in maintaining historical genetic connectivity among neighboring populations. Additionally, our ENMs demonstrate that these regions consistently exhibited high habitat connectivity in the past, with these persistently suitable habitats acting as key ecological corridors that have effectively facilitated population dispersal and gene flow.

Recent demographic inferences for the N, C, and SW clusters revealed repeated fluctuations in *N*_e_ over the past 200 generations. Although identifying precise drivers remains challenging, all three populations showed consistent population declines over ∼300 years, probably due to human expansion, agricultural expansion into the mountains, overhunting for food/traditional medicine, and habitat destruction from forest exploitation (Yang 1988; Long et al. 1994; Zhao et al. 2018). Historical records indicate these pressures intensified during the Ming and Qing dynasties when state-sponsored migrations brought Han Chinese settlers to southwest China (Liu et al. 2009), introducing farming technologies and causing human expansion that probably drove declines and local extinctions of *R. bieti*. Notably, populations in the southern and central regions began their recent declines significantly earlier than those in the northern areas. We hypothesize that this temporal pattern may reflect earlier exposure of southern and central populations to human pressures, consistent with the earlier establishment of human settlements and agricultural activities. Furthermore, compared to predecline levels, the *N*_e_ of central and southern populations has plummeted by nearly 98%, indicating substantially greater human-induced pressure. This inferred change aligns with contemporary spatial distribution patterns of road networks and human settlements. In addition, while complex genetic admixing in SE and NC clusters complicates recent demographic history analysis, their low genetic diversity suggests severe bottlenecks in the past attributable to overexploitation or founder effects.

Based on our large-scale population genomic study, we recommend the following management measures to maintain genetic connectivity and diversity in *R. bieti* populations.

- Results of population genetic structure analysis revealed that *R. bieti* can be divided into five genetically distinct clusters, with each cluster isolated in a relatively independent habitat patch. Contemporary gene flow analysis also detected no significant migration signals, leading us to recommend designating these five genetic clusters as separate conservation units for differentiated conservation. Although previous studies using ecological and landscape genetic approaches suggested the presence of migration corridors (Li et al. 2015b), these corridors appear insufficient to restore effective gene flow between the five major genetic clusters. Therefore, we recommend prioritizing the enhancement of habitat connectivity within conservation units by establishing ecological corridor networks between core habitats and high-density populations. This natural dispersal-based gene flow maintenance strategy could effectively preserve the genetic diversity characteristics of each cluster while avoiding potential risks associated with artificial translocation, and would help maintain ecological connections between core and peripheral populations.
- Based on our genomic, landscape genetic, and ecological niche modeling analyses, we propose that the southwest conservation unit should be given the highest conservation priority. This conclusion is supported by two primary lines of evidence: (i) A unique but vulnerable evolutionary lineage: The southwest unit represents a significantly distinct genetic lineage, indicating a unique evolutionary trajectory. However, this distinctiveness coincides with a relatively low level of genetic diversity, characterizing it as a nonreplaceable yet highly vulnerable component of the species’ overall genetic diversity. (ii) Severe and multifaceted threats: Our assessments reveal that the southwest unit faces the most intense pressures from human activities and climate change. This is reflected in it having the highest degree of habitat fragmentation and the lowest landscape connectivity of all the units. Furthermore, our ecological niche modeling confirms that its suitable habitat has exhibited, and continues to show, high sensitivity to climatic oscillations. Therefore, while we maintain that enhancing habitat connectivity within all identified clusters is important, prioritizing targeted conservation actions for the southwest unit is essential. This strategy is crucial to preventing the irreversible loss of its unique evolutionary lineage and mitigating its acute vulnerability.
- From a macro perspective of *R. bieti* conservation, wildlife managers need to carefully evaluate the impact of human activities in areas with low genetic connectivity and variation to ensure these activities do not further exacerbate the decline of vulnerable populations. In areas with restricted connectivity, continued intensification of human activities may further disrupt natural population exchanges, leading to the further isolation of small populations. Targeted measures in these areas would significantly improve local connectivity and maintain long-term genetic diversity and adaptive potential.
- According to the international conservation standard of the “50/500 rule” (Jamieson and Allendorf 2012; Frankham et al. 2013, 2014; Caballero et al. 2017), when *N*_e_ falls below 500, isolated populations will suffer inbreeding depression and genetic diversity loss. The short-term conservation target should maintain *N*_e_ ∼50, while long-term species survival requires *N*_e_ > 500. Our data show evidence of extremely low *N*_e_ (∼40 to 314), making the species potentially vulnerable to future ecological changes and human activity pressures. Thus, we recommend enhancing gene flow by promoting effective migration in each generation.
- Given the present isolation of local groups and the potential risk of intensified genetic drift, it is essential to implement regular genetic monitoring to track dynamic changes in genetic variation and structure. Based on our landscape connectivity and landscape genetic analyses, roads and human settlements were detected as the primary barriers to contemporay gene flow. Future conservation efforts would prioritize tracking the monkey groups’ movement and monitoring the impact of these barriers on them. In addition, the wildlife corridors can be established to reconnect habitats. For example, the land-use planning, such as avoiding new road construction in core activity areas and, where feasible, relocating settlements, can help restore population connectivity.

In summary, we first provide a comprehensive analysis of contemporary spatial patterns of population structure in *R. bieti* and then reconstruct the species’ demographic history through the use of whole-genome SNP data and the inclusion of previously unsampled monkey groups. A deeper understanding of the genetic status and evolutionary processes will clarify extinction risks and refine genetic management strategies for fragmented populations. Given ongoing human pressures and accelerating habitat fragmentation in this species’ range, we recommend establishing a framework for regular reassessment of conservation units.

## Materials and methods

### Sampling and DNA extraction

Long-term, dedicated sampling efforts allowed us to obtain a total of 494 fecal samples from 20 *R. bieti* groups (Fig. 1; Table S1). To avoid resampling the same individual, each dropping was distinguished by freshness, size, shape, and color, and feces found less than 1.5 m apart were not sampled (Hayaishi and Kawamoto 2006; Liu et al. 2007). All fecal samples were stored in the field and preserved in 100% ethanol or fecal DNA preservation solution (Cat. No. 4111050, SIMGEN, Zhejiang, China). Fecal total DNA extractions were performed in the laboratory at the Yunnan University using the QIAamp Fast DNA Stool Mini Kit (Cat. No. 51604, QIAGEN, Germany) following the manufacturer's instructions. Briefly, a 200 to 500 µL sample of 95% ethanol or DNA preservation solution mixture was resuspended in InhibitEX buffer, clarified by centrifugation, treated with proteinase K, and passed through a DNA binding column. Bound DNA was finally eluted in 50 to 80 µL elution buffer. DNA quality and quantity were assessed on 1% agarose gels and on a Qubit Fluorometer, respectively.

### Targeted region selection, capture, and sequencing

We used target capture sequencing (TargetSeq), a cost-effective next-generation sequencing method suitable for low-quality DNA from fecal samples, to enrich targeted genomic regions and detected genetic variation (Jones and Good 2016). This method reduces sequencing costs and improves the quality of the data by hybridizing DNA libraries with complementary baits, thereby increasing the proportion of on-target DNA (Fontsere et al. 2021, 2022; Ozga et al. 2021). To design a TargetSeq panel of targeted regions, we selected potentially neutral variation regions across 35 *R. bieti* genomes based on a population-level SNP dataset reported in Wu et al. (2023). The putatively neutral regions were selected as follows: (i) We excluded sites located within annotated gene regions; (ii) We also removed sites located within CpG island regions, as such regions are known to exhibit elevated mutation rates and may be subject to selective pressures (Johri et al. 2021; Soni and Jensen 2025). We further filtered this dataset to retain loci that were successfully genotyped in all *R. bieti* individuals and had a minor allele frequency (MAF) of at least 0.05 and a Hardy–Weinberg equilibrium with *P* < 0.001, potentially leading to false positives caused by sequencing errors or genotype-calling inaccuracies (Hemstrom et al. 2024). The retained loci were further thinned to one SNP per 10 kb to reduce the possibility of linkage. Only biallelic autosomal SNPs were retained (i.e. triallelic SNPs and SNPs with mapping to the X/Y chromosomes were excluded). This resulted in candidate regions with 54,892 retained autosomal SNPs, which were randomly distributed across each chromosome of the whole genome (Table S7).

Based on candidate neutral regions, we further designed and synthesized target nucleotide-long single-stranded RNA biotinylated capture probes for capturing and sequencing fecal DNA of 494 *R. bieti* individuals. To prepare DNA sequencing libraries, approximately 300 ng of genomic fecal DNA from each sample was sheared by iGeneTech to obtain 150 to 200 bp fragments. The ends of DNA fragments were repaired, and Illumina adapters were added (IGT Enzyme Plus Library Prep Kit, iGeneTech, Beijing, China). The probe hybridization, target capture, post-capture amplification, and bead cleanup of captured amplified DNA are described in Li et al. (2025). Captured libraries were sequenced on the Illumina Novaseq 6000 platform with 150 bp paired-end (PE) reads. The probe synthesis, library preparation, and sequencing were performed by iGeneTech (Beijing, China).

### SNP genotyping and filtering

Data quality of the Illumina raw reads was initially assessed using Fastp (Chen et al. 2018). Adapters and low-quality bases were removed with Fastp using the parameters “-W 4 -M 20 -n 0 -q 20 -5 5 -3 5.” The resulting clean PE reads were then aligned to the de novo assembled *R. bieti* reference genome (Wu et al. 2023) using the BWA-MEM (Li and Durbin 2009) algorithm with default settings. SAMtools (Li et al. 2009) was used to sort the resulting BAM files. These sorted BAM files were subsequently merged, coordinate-sorted, and deduplicated using Picard v2.10.3 (http://picard.sourceforge.net) with the MergeSamFiles, SortSam, and MarkDuplicates tools, respectively. Finally, insertion/deletion (INDEL) realignment was performed using the GATK (v4) (Mckenna et al. 2010) IndelRealigner tool.

A target file of 54,892 SNP positions was analyzed using GATK's UnifiedGenotyper to generate a VCF file. To obtain a high-quality and credible SNP dataset, we first excluded nucleotides with a base phred quality score < 20 or those located in reads with a mapping phred quality score < 20. The raw callset was then filtered by excluding variants matching at least one of the following criteria: nonbiallelic SNP; a SNP phred quality score (QUAL) < 60; a significant fisher strand test (FS > 60); a variant confidence/quality by depth (QD) < 2; a root mean square of the mapping quality (MQ) < 40; a MQRankSum < −20 or a significant read position bias (ReadPosRankSum < −8.0). Subsequent filtering in VCFtools (Danecek et al. 2011) excluded variants with MAF < 0.05 and SNPs missing in > 80% of all samples. In addition, we assessed the mean depth per site and missing rate for each individual. We excluded 164 individuals due to low depth (mean depth < 4-fold) or high missing rates (missing rate > 30% of genotypes across all target sites).

To avoid potential bias from closely related individuals, we used PLINK2 software (https://www.cog-genomics.org/plink/2.0/) with the “make-king” command to calculate kinship coefficients for all pairs of individuals and excluded 21 individuals with first-degree relationships (i.e. a kinship coefficient > 0.177) from our dataset. Following these filtering steps, 309 *R. bieti* individuals remained for subsequent analysis (Table S1).

### Phylogenetic relationships and population structure analysis

To visualize the population structure of *R. bieti*, we conducted phylogenetic reconstruction, PCA, and ADMIXTURE analysis (Alexander et al. 2009). For phylogenetic analysis, we first used the genetic information at individual and population levels, and included three golden snub-nosed monkey (*R. roxellana*) individuals as the outgroups (Table S1; Wu et al. 2023). At the individual level, an NJ tree was constructed using MEGA-X (Kumar et al. 2018) software with the p-distance model. In total, 1,000 bootstraps were performed. An ML tree was then generated using RAxML (v8) (Stamatakis 2014 ) under the GTRGAMMA model with 1,000 bootstrap replicates. Visualization was performed in Figtree (http://tree.bio.ed.ac.uk/software/figtree/). At the population level, we ran TreeMix (Pickrell and Pritchard 2012) based on geographic monkey groups to infer patterns of past population divergence and included three golden snub-nosed monkeys as the outgroups. TreeMix returns an ML tree based on the allele frequency covariances for the given populations.

Second, PCA was performed using the smartPCA program in the Eigensoft package (Price et al. 2006) without outlier iterations. The Tracy–Widom test was used to determine the significance level of the principal components. The score for the first component (PC1) was plotted against the score for the second component (PC2) for all individuals to illustrate the genetic affinity between all samples. To detect substructure within all populations, we conducted additional PCA on population subsets. All PCA results were visualized in R.

Third, we used a clustering model in ADMIXTURE (v1.3.0) (Alexander et al. 2009) to assess genetic structure across 20 monkey groups. This model uses multilocus genotypes to estimate the ancestry proportions of each individual in each ancestral population (*K*). To eliminate the clustering bias resulting from different sample sizes, we randomly selected the same number of individuals from each monkey group for ADMIXTURE analysis. For this analysis, we simulated *K* from 1 to 20, representing the total number of sampling localities. For each value of *K*, we conducted 200 replicate runs with 10 iterations, and identified the most well-supported number of genetic clusters based on the lowest CV error value. Admixture plots were generated using the Admixture Q Matrix Viz tool in TBtools (Chen et al. 2020) software.

### Genetic differentiation, isolation by distance, and genetic connectivity

To quantify the differentiation between populations, we calculated pairwise genetic differentiation (*F*_ST_) between all monkey groups and five genetic clusters using the population module of Stacks (v2.62) (Catchen et al. 2013). Subsequently, we examined potential signatures of IBD based on least cost paths. IBD describes the correlation between increasing genetic divergence and geographic distance, reflecting limitations in dispersal across landscapes. Before IBD analysis, *F*_ST_ values were transformed to a continuous scale as genetic distance using the formula *F*_ST_/(1 − *F*_ST_). The geographical coordinates of the central point of each population were used to calculate the geographic distance between them. We then tested for a statistically significant correlation between genetic distance and the geographic distance using a Mantel test implemented in the R package.

To visualize population structure and estimate gene flow across the sampled areas, we used EEMS (Petkova et al. 2016). We generated a distance matrix from our SNP dataset using the program “bed2diff_v1” provided with EEMS. We ran independent chains from different starting seeds for 2 × 10^7^ MCMC iterations, with a burn-in of 2 × 10^6^ iterations and a thinning rate of 5,000 iterations. We specified a deme size of 500 for the analysis. We assessed the convergence of the MCMC using a posterior probability trace plot. EEMS generates maps showing a pattern of relative effective mobility (*m*) centered on mean values. Regions exhibiting higher-than-average genetic differentiation appear as deviations below the mean, indicating potential barriers to gene flow, while regions below the mean suggest corridors facilitating gene flow. The resulting EEMS output was visualized using the “make_eems_plots” R script (available at https://github.com/dipetkov/reemsplots2).

We also estimated contemporary migration rates and direction using a Bayesian identification of first- and second-generation immigrants in BA3-SNPs (Wilson and Rannala 2003; Mussmann et al. 2019). Acceptance rates for the MCMC chain were optimized to 28% to 50% by adjusting mixing parameters with BA3-SNPs-autotune, and convergence was confirmed by log probability traces in Tracer (v1.7) (Rambaut et al. 2018). Final estimates were obtained in a run with 15 million MCMC iterations, with a burn-in period of 5 million and a sampling interval of 100 iterations.

### Landscape genetic analysis

To evaluate the influence of landscape variables on connectivity and genetic discontinuities in *R. bieti* populations, we conducted the landscape connectivity and landscape genetic analyses. We used 11 landscape variables relevant to *R. bieti* movement: seven natural environmental factors (elevation, slope, aspect, temperature, precipitation, waterways, and vegetation) and four human variables (roads, human settlements, human population density, and land use; Table S2; Figure S10). These variables are consistent with earlier connectivity studies of this species (Shi et al. 2025). Using MaxEnt (v3.4.4) (Phillips et al. 2006) and ArcGIS (https://www.arcgis.com/), we generated a resistance surface across the species’ habitat. The geographical occurrence records (*n* = 77) were derived from field surveys in this study (*n* = 41) and a previously published dataset (*n* = 36). Duplicated records and those outside the species’ current distribution range were removed. To avoid spatial sampling bias, randomized spatial thinning was performed on all datasets using the R package spThin (Aiello-Lammens et al. 2015) with 100 iterations, enforcing a minimum distance of 1.5 km between observations. This resulted in 50 spatially filtered occurrence points for connectivity analysis. To construct the resistance surface, we first converted the habitat suitability ASCII file output from the MaxEnt model into raster format and defined its coordinate system as WGS 1984. Subsequently, using the raster calculator tool in ArcGIS, resistance values were assigned based on the difference between the theoretical maximum suitability and the suitability value of each pixel. Finally, a landscape resistance surface was generated based on these assigned resistance values.

Landscape resistance surfaces were parameterized by ResistanceGA (Peterman 2018). Specifically, the algorithm maximizes the correlation between pairwise genetic distances (*F*_ST_ as response variables) and pairwise landscape distances (including Euclidean distance as a predictor). Surface optimization was performed individually using the *commuteDistance* function from the *gdistance* package in R, with three independent runs to ensure parameter convergence. We used the *resist.boot* function for bootstrapping within the ResistanceGA package with 1,000 iterations. We randomly selected 75% of the samples without replacement and fitted each surface to each sample subset. The optimal univariate and multivariate models were selected based on the lowest Akaike's Information Criterion (AIC) score.

### Genetic diversity

We characterized genetic diversity by calculating observed heterozygosity (*H*o) for each individual using the population module in Stacks (v2.62) (Catchen et al. 2013) software. To visualize genetic diversity across the sampled areas, we also used EEMS to reveal patterns of spatial distribution of genetic diversity (see Methods). EEMS generates maps showing relative genetic diversity (*q*) centered on the mean, allowing high and low levels of genetic diversity to be visualized. To minimize the influence of sample variation on the results, we employed a random sampling approach, conducting 200 iterations and selecting an equal number of individuals from each population for all genetic diversity.

### Recent demographic history

We inferred demographic history using GONE (Santiago et al. 2020), which estimates the effective population size (*N*_e_) for generations back from the present on the basis of the relationship between SNP pairs in LD and distance in the chromosome. Given GONE's limitations in admixed populations (Novo et al. 2023), we applied it only to the least admixed populations, that is, the SW, C2, and N lineages. Our parameters for GONE followed the default settings and assumed a constant recombination rate of 1 cM/Mb across the genome. We ran 40 iterations of GONE, randomly excluding 10% of individuals per run (McCarthy et al. 2025). The mean *N*_e_ and 95% CIs were calculated and visualized in R.

### Model fitting using Fastsimcoal2

To identify dispersal patterns best explaining the observed genetic structure, we used an approach implemented in Fastsimcoal2 (Excoffier et al. 2021) that estimates a composite-likelihood based on coalescent simulations in order to fit a demographic model and associated parameters to the observed site frequency spectrum (SFS). The SFS was generated using easySFS (https://github.com/isaacovercast/easySFS). Based on phylogenetic relationships and genetic diversity patterns, we first evaluated two alternative demographic hypotheses: a possible southwestern origin of *R. bieti* (Figure S7a); a possible central origin of *R. bieti* (Figure S7b). Based on the optimal model identified in this initial comparison, we subsequently tested six dispersal scenarios: stepping-stone dispersal model (Model 1; Figure S8a), stepping-stone with admixture origin of SE model (Model 2; Figure S8b), stepping-stone with admixture origin of NC model (Model 3; Figure S8c), stepping-stone with admixture origin of SE/NC model (Model 4; Figure S8d), isolation-with-migration model (Model 5; Figure S8e), and independent migration model (Model 6; Figure S8f). All *N*_e_ parameters were set to be constant over time and were drawn from a uniform distribution with range [50, 1 × 10^4^]. We simulated three historical divergence events for the stepping-stone model in which each divergence time parameter was drawn from a uniform distribution with range [100, 5 × 10^4^]. We bounded the lower range of each divergence event by the preceding divergence event to simulate a stepping-stone model. Parameter estimates for each migration rate were drawn from a uniform distribution with range [10^−6^, 10^−2^]. When fitting our observed data, we performed 100,000 simulations per parameter set across 40 conditional maximization cycles (following Fastsimcoal2 recommendations). Each model was independently run 100 times with unique random seeds to avoid local likelihood maxima and ensure convergence to the global optimum. The best run for each model was selected based on maximum estimated likelihood, followed by model comparison using AIC. The mutation rate per generation was set to 1.36 × 10^−8^, and with a generation time of 10 years (Zhou et al. 2016).

To further investigate the recent divergence history among subpopulations, we performed demographic simulations using Fastsimcoal2 with the same parameter settings, modeling the divergence, gene flow, and *N*_e_ within the four genetic clusters (SW, C, NC, and N; Fig. 6b to e).

### Modeling past range dynamics

To investigate the distributional dynamics of *R. bieti* in response to past climate changes, we constructed ENM using MaxEnt (v3.4.4) (Phillips et al. 2006). Fifty spatial occurrence points were used to construct the ENM. We built ENMs for four time periods: the LIG (∼129 to 116 ka), LGM (∼27 to 19 ka), MH (∼8.2 to 4.2 ka), and the present day (1970 to 2000 AD). Nineteen bioclimatic variables were downloaded from WorldClim (www.worldclim.org). To mitigate redundancy among the climate layers, we calculated pairwise Spearman correlation coefficients and iteratively removed variables exceeding a threshold of |*r*| > 0.7 using ArcGIS v10.8 Resample Data Management Tools. This resulted in four retained variables for the final MaxEnt models: mean diurnal range (BIO2), isothermality (BIO3), max temperature of warmest month (BIO5), and temperature annual range (BIO7) (Table S8 and Figure S11). The final MaxEnt runs were conducted with a 25% testing ratio and a minimum training presence logistic threshold, and the other parameters set to default values. The model performance was evaluated using the AUC. We generated ensemble projections for each period from the MaxEnt outputs, calculated as the weighted mean of predicted presence probabilities. Final distribution maps were plotted using ArcMap v10.8 (https://www.arcgis.com/).

## Supplementary Material

msag104_Supplementary_Data

## Data Availability

Raw sequencing data are available from the China National GeneBank DataBase (CNGBdb) under accession number (PRJCA043696). Code for data processing and analysis, and simulations used in this study have been deposited in GitHub (https://github.com/weiminKuang/Project_for_R.bieti).
