# Supplementary material for: Genome-wide SNP data reveal hidden hierarchical population structure and demographic history of endangered black-and-white snub-nosed monkeys (Rhinopithecus bieti)
Source: Mol Biol Evol. 2026 Apr 17;43(5):msag104. doi: 10.1093/molbev/msag104 (PMC13159727; doi:10.1093/molbev/msag104)
Supplement: msag104_Supplementary_Data [file msag104_supplementary_data.pdf]

Supplementary Materials

Genome-wide SNP data reveal hidden hierarchical population structure and demographic history of endangered black-and-white snub-nosed monkeys (*Rhinopithecus bieti*)

Yitian Li[1,†], Zhiru Xu[1,†], Yingli Jiang[1,†], Minglin Chen[1], Yuan Li[1], Fan Liu[1], Jia Luo[1], Jiachao Feng[1], Weimin Kuang[1,*], Li Yu[1,2,*]

[1]School of Life Sciences, State Key Laboratory for Conservation and Utilization of Bio-Resource in Yunnan, Yunnan University, Kunming 650500, China
[2]Southwest United Graduate School, Kunming 650500, China

[†]These authors contributed equally to this work.

***Correspondence author:** Li Yu (Email: yuli@ynu.edu.cn); Weimin Kuang (Email: kuangwm0714@ynu.edu.cn).

Figure S1. A population-level ML tree constructed using the TreeMix method. Groups were categorized into five distinct genetic clusters: Northern (N; red), North of central (NC; dark green), Central (C; blue), Southeastern (SE; purple), and Southwestern (SW; orange).

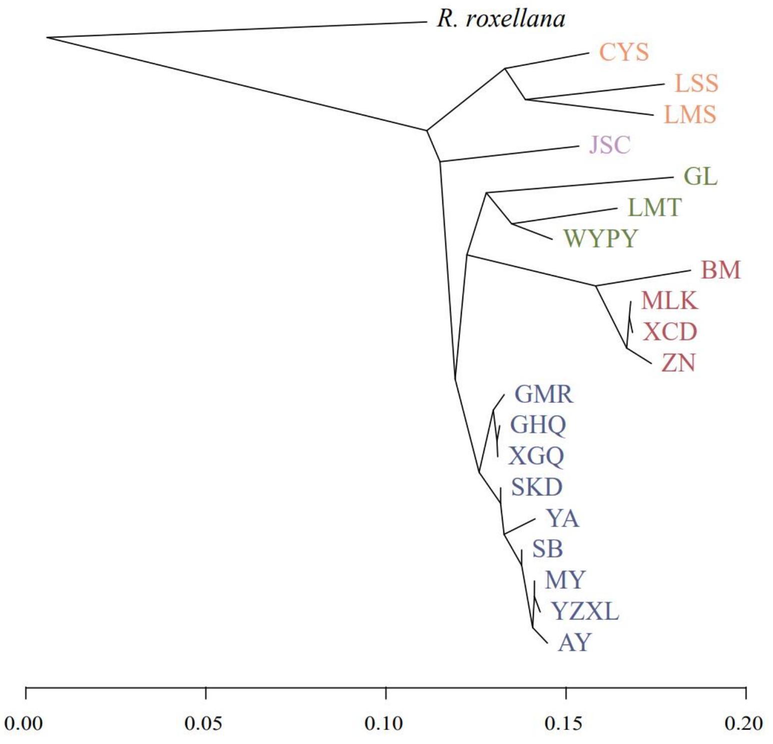

Figure S2. Principal component analysis (PCA) of five populations based on varying random sample sizes. PCAs were performed by randomly selecting the same sample size from each population with varying gradients, including 5 individuals (A), 10 individuals (B) and 15 individuals (C). Samples from different populations (clusters) are represented by different colors: N (red), NC (dark green), C (blue), SE (purple), and SW (orange).

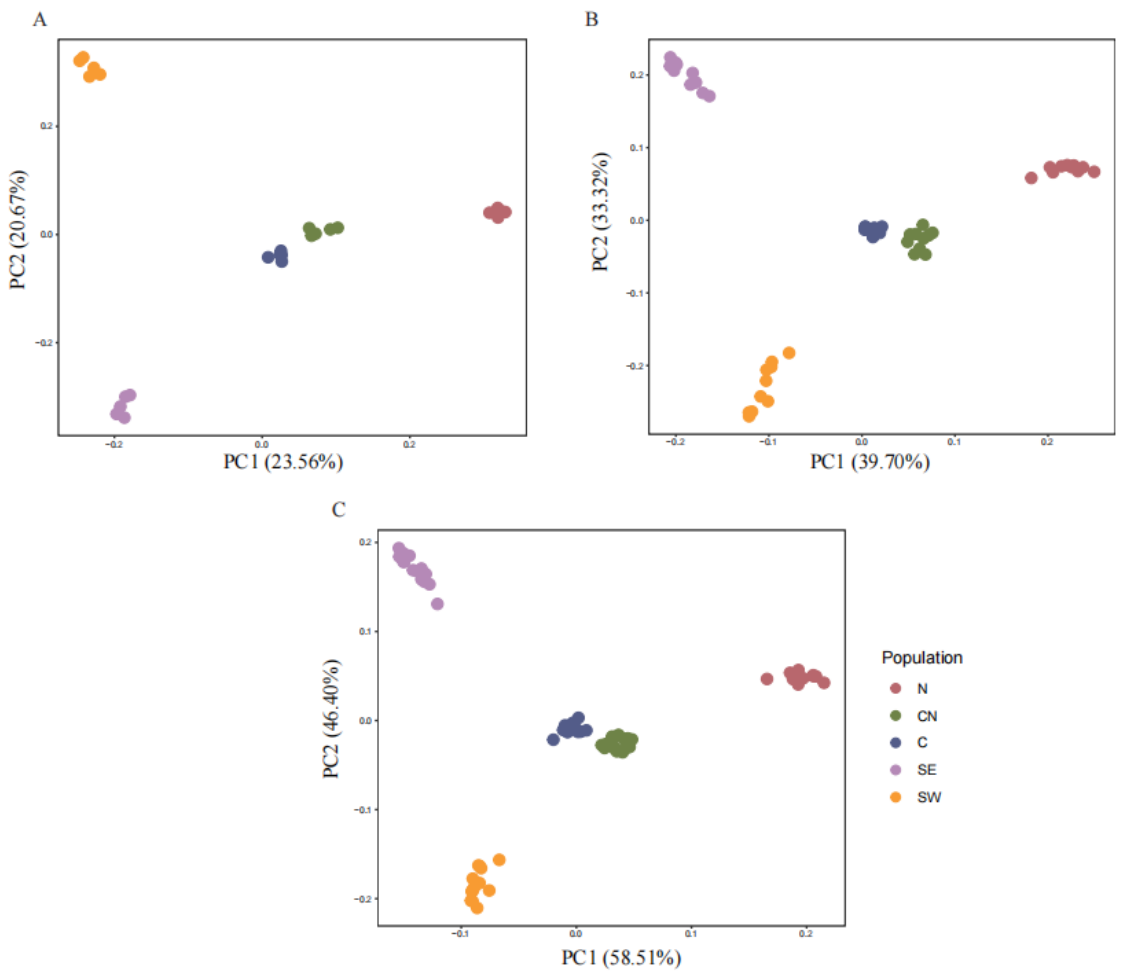

Figure S3. Cross-validation (CV) error values in the ADMIXTURE analysis. The red horizontal line represents the minimum CV error, which occurs at K = 4. However, similar CV error values were also obtained for K=3 and 5.

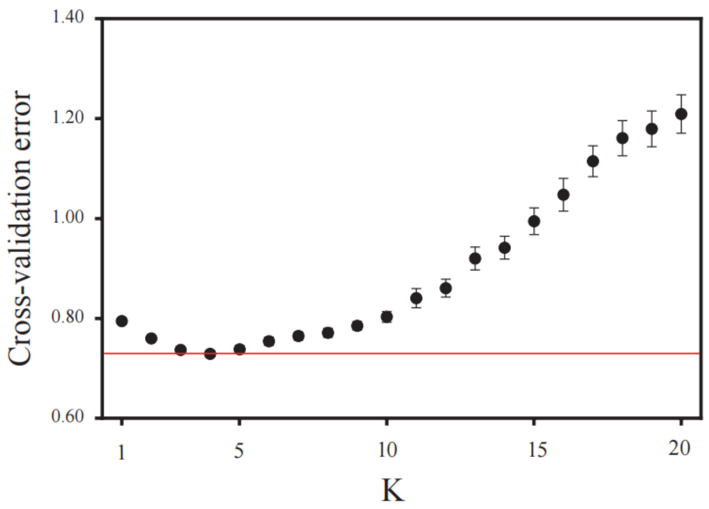

Figure S4. Pairwise genetic differentiation (F_{ST}) values between 20 monkey groups. Each square represents the genetic distance between two groups, with red colors indicating higher F_{ST} values, and blue colors representing lower F_{ST} values.

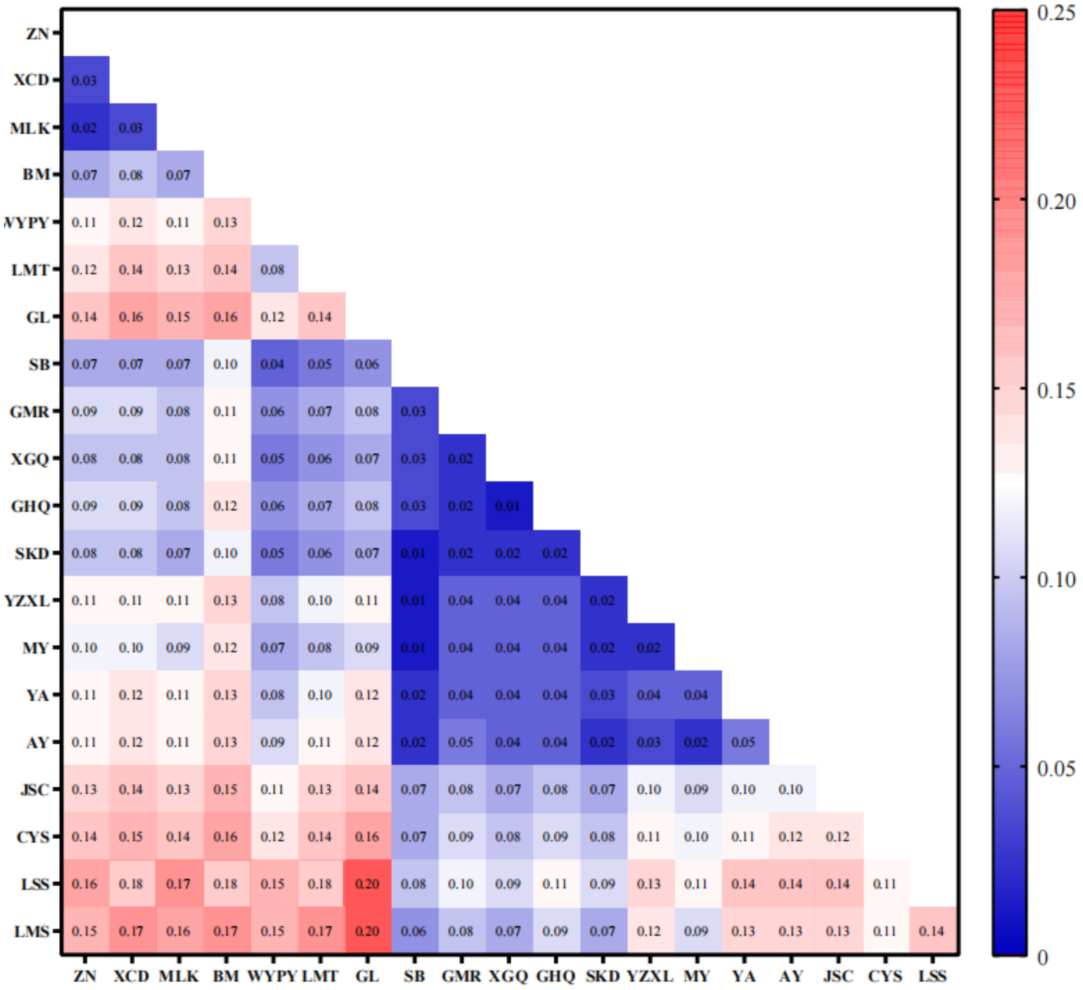

Figure S5. Observed heterozygosity (*H*o) across five populations based on 200 random samples. The bar plot shows the distribution of *H*o for five populations (N, NC, C, SE, and SW), which was calculated from 200 independent sampling.

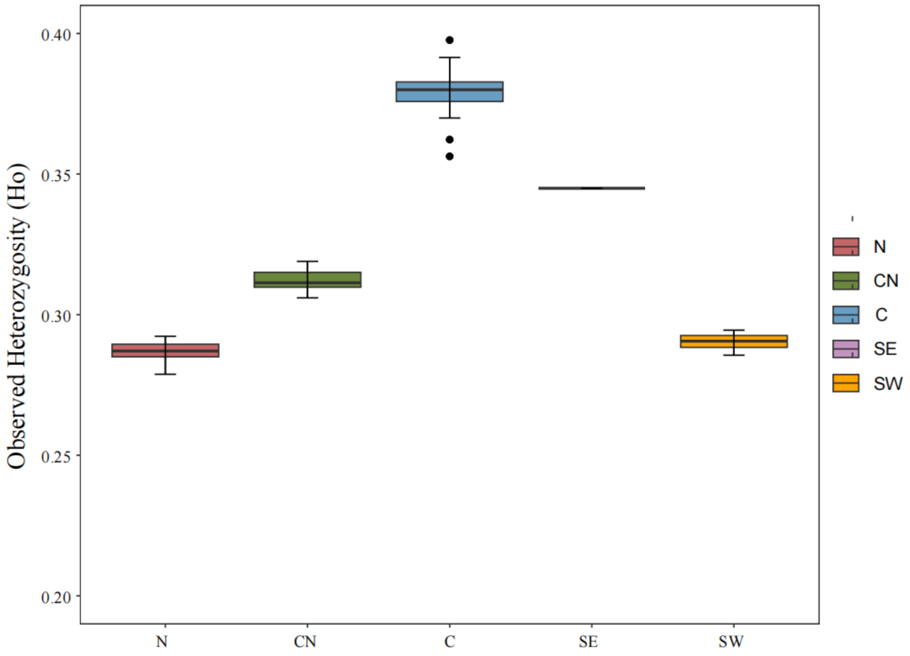

Figure S6. Correlation between observed heterozygosity (*H*o) and census population size. Each point represents a group.

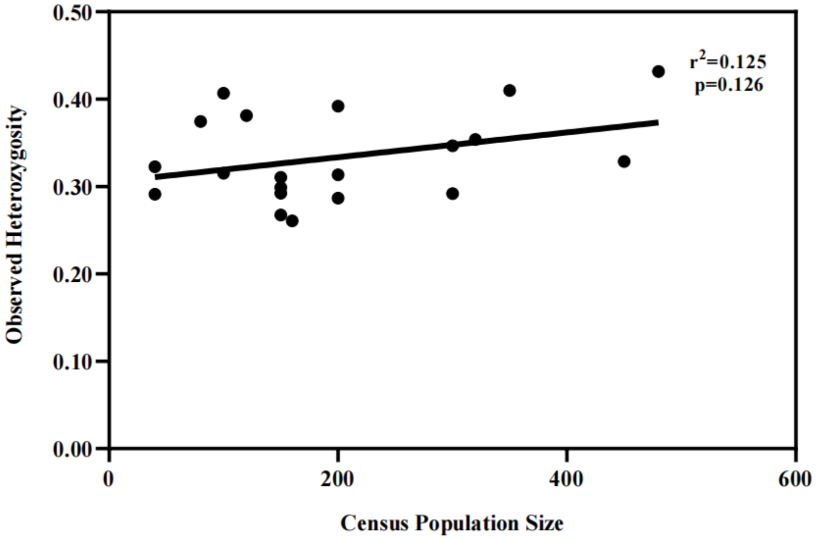

Figure S7. Demographic scenario test in Fastsimcoal2: (A) A possible southwestern (SW population) origin of *R. bieti*; (B) A possible central (C population) origin of *R. bieti*.

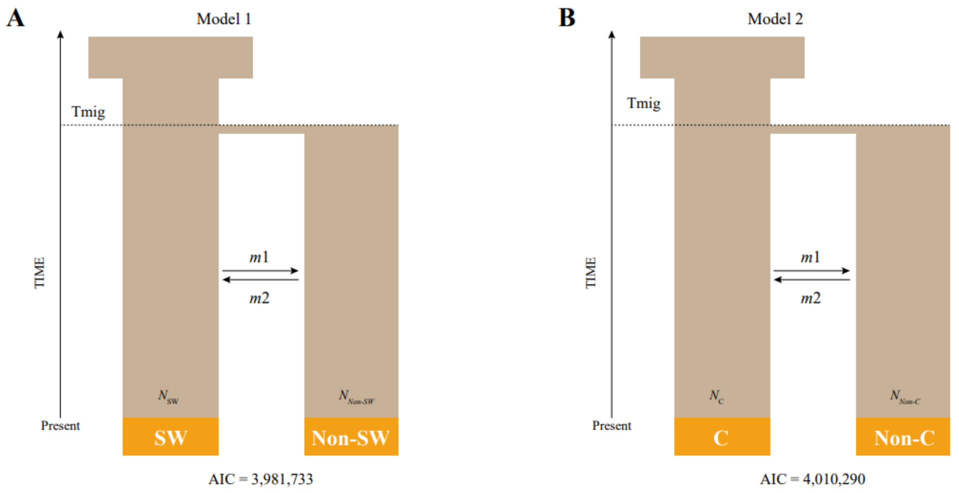

Figure S8. Schematics of demographic models using Fastsimcoal2: (A) Model 1: Stepping-stone dispersal model; (B) Model 2: Stepping-stone dispersal with admixture origin of SE model; (C) Model 3: Stepping-stone dispersal with admixture origin of NC model; (D) Model 4: Stepping-stone dispersal with admixture origins SE/NC model; (E) Model 5: Isolation-with-migration model; (F) Model 6: Independent migration model.

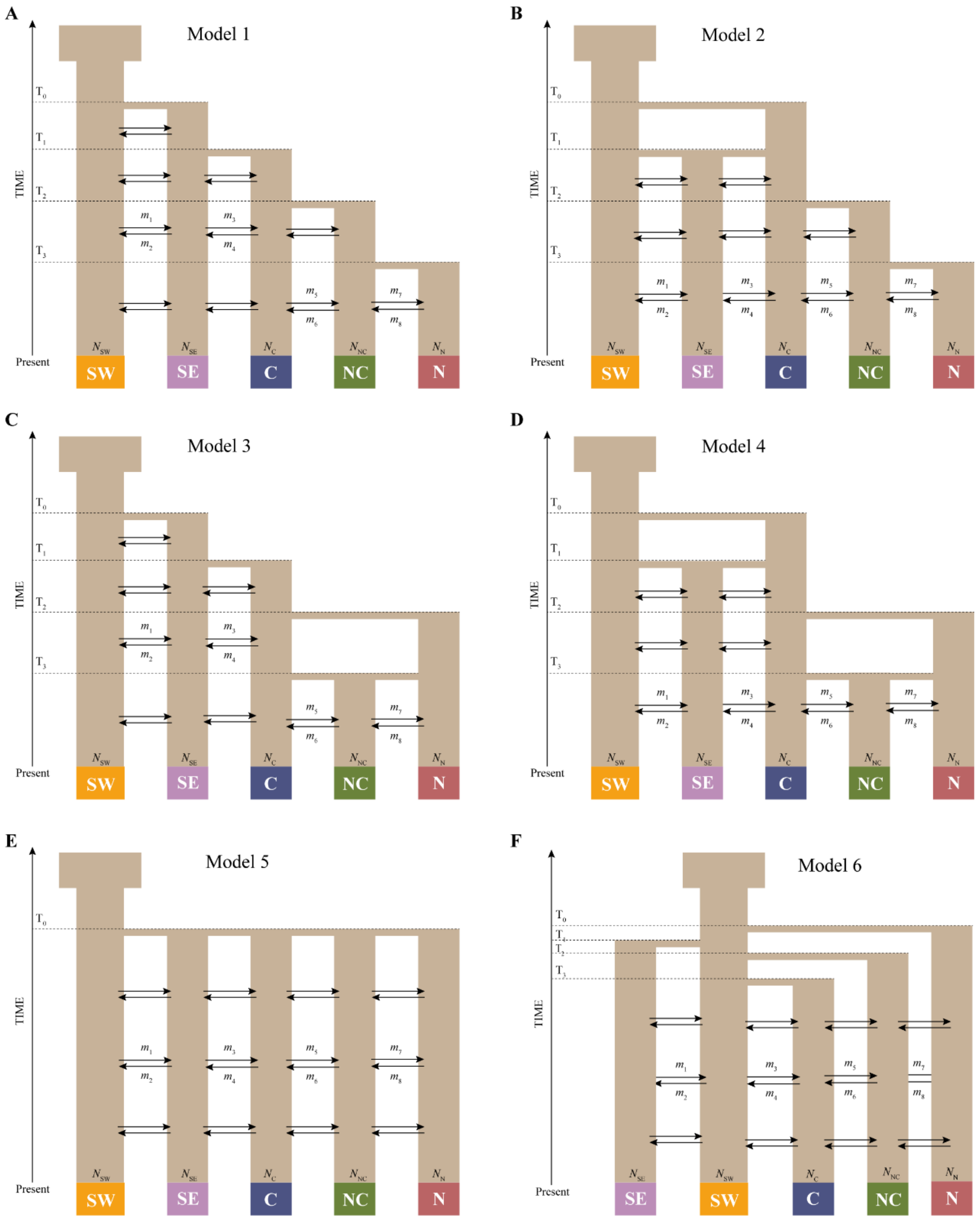

Figure S9. The observed and expected 2D- (A) and 1D-SFSs (B) for the best-fitting inferred demographic model. The R script SFStools (https://github.com/marqueda/SFS-scripts) was used to visualize and compare the observed and expected 2D- and 1D-SFSs.

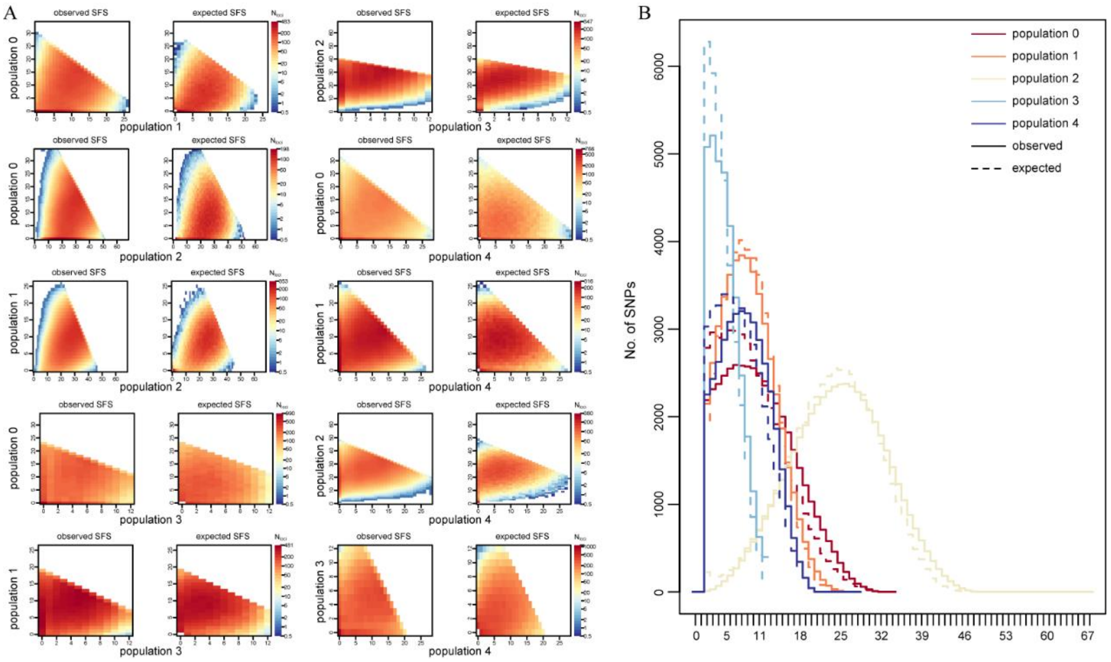

Figure S10. Pearson correlation coefficients for the eleven landscape variables.

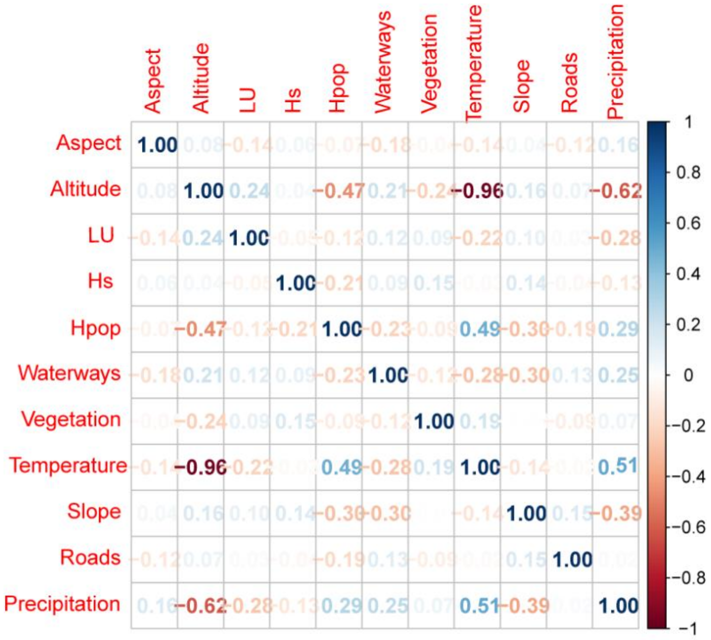

Figure S11. Pearson correlation coefficients for the 19 bioclimatic variables.

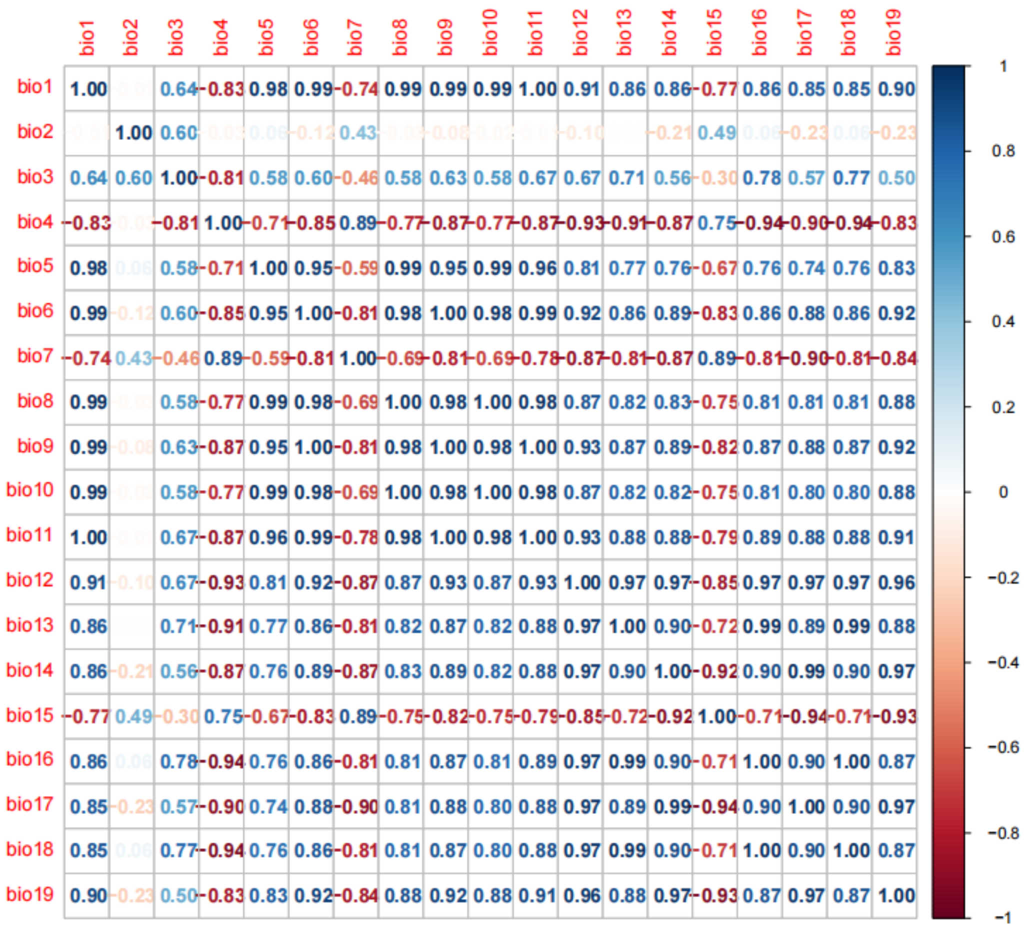

Table S1. Sample information and sequencing data summary for 494 *R. bieti* individuals and the three *R. roxellana* individuals that were used as outgroups in this study.

Sample ID	Identified genetic cluster	Coverage > 1-fold (%)	Average depth (%)
ZN-01	N	98.58%	20.25
ZN-02	N	96.69%	12.70
ZN-03	N	99.83%	94.42
ZN-04	N	88.80%	7.16
ZN-05	N	99.66%	49.53
ZN-06	N	98.22%	17.79
ZN-07	N	99.91%	175.66
ZN-08[a]	N	52.72%	3.12
ZN-09	N	99.34%	28.61
ZN-10	N	99.58%	37.99
ZN-11	N	78.65%	6.74
ZN-12	N	99.66%	49.36
ZN-13	N	98.36%	20.31
ZN-14	N	87.64%	7.02
ZN-15	N	95.80%	11.11
ZN-16[a]	N	51.94%	2.95
ZN-17	N	99.51%	36.20
ZN-18[a]	N	25.62%	3.94
ZN-19[a]	N	73.33%	3.97
XCD-01	N	82.33%	4.72
XCD-02[a]	N	21.49%	2.02
XCD-03	N	93.40%	7.89
XCD-04	N	97.61%	18.86
XCD-05	N	89.15%	6.30
XCD-06	N	96.56%	13.37
XCD-07[a]	N	74.87%	4.10
XCD-08[a]	N	51.24%	2.96
XCD-09[a]	N	57.99%	3.03

XCD-10[a]	N	25.84%	2.11
XCD-11[a]	N	63.54%	5.15
XCD-12	N	99.82%	91.71
XCD-13[a]	N	99.36%	30.20
XCD-14[a]	N	99.34%	27.01
XCD-15[a]	N	99.84%	92.23
XCD-16	N	99.86%	122.71
XCD-17	N	99.03%	22.56
XCD-18[a]	N	26.39%	2.43
XCD-19	N	99.22%	25.68
XCD-20	N	98.59%	18.75
XCD-21[a]	N	99.92%	231.09
XCD-22[a]	N	99.74%	53.38
XCD-23	N	99.83%	83.74
XCD-24	N	99.55%	36.31
MLK-01	N	98.23%	16.02
MLK-02[a]	N	99.46%	34.91
MLK-03	N	99.47%	38.85
MLK-04[a]	N	48.33%	2.78
MLK-05	N	96.28%	11.69
MLK-06	N	99.03%	23.54
MLK-07	N	99.41%	30.17
MLK-08	N	99.81%	87.47
MLK-09	N	99.93%	279.52
MLK-10	N	91.50%	8.02
MLK-11	N	99.87%	113.68
MLK-12[a]	N	51.27%	2.94
MLK-13[a]	N	55.61%	3.29
MLK-14	N	97.60%	13.41
MLK-15	N	99.76%	65.57
MLK-16[a]	N	74.52%	4.26
MLK-17[a]	N	56.60%	3.17
MLK-18[a]	N	29.96%	2.18
MLK-19[a]	N	52.60%	2.88

MLK-20[a]	N	20.22%	3.05
BM-01	N	99.04%	24.48
BM-02[a]	N	99.71%	57.97
BM-03	N	97.37%	13.22
BM-04	N	95.63%	10.37
BM-05	N	87.77%	5.98
BM-06	N	82.68%	4.85
BM-07	N	88.88%	6.25
BM-08	N	88.48%	6.30
BM-09	N	92.90%	7.72
BM-10	N	99.57%	39.68
BM-11[a]	N	99.21%	25.77
BM-12	N	98.99%	24.40
BM-13	N	99.14%	24.58
BM-14	N	98.91%	23.22
BM-15	N	99.24%	24.52
BM-16	N	95.61%	10.79
BM-17	N	97.18%	14.04
BM-18	N	98.75%	19.32
BM-19	N	87.13%	6.23
BM-20	N	98.49%	17.77
BM-21[a]	N	71.81%	4.29
BM-22	N	99.63%	44.73
WYPY-01	NC	83.35%	5.21
WYPY-02	NC	98.87%	22.70
WYPY-03	NC	79.54%	4.70
WYPY-04	NC	98.99%	23.98
WYPY-05[a]	NC	21.19%	2.08
WYPY-06	NC	98.41%	21.29
WYPY-07[a]	NC	55.96%	3.06
WYPY-08[a]	NC	64.90%	3.55
WYPY-09[a]	NC	96.53%	10.92
WYPY-10[a]	NC	71.98%	3.89
WYPY-11	NC	98.90%	20.95

WYPY-12[a]	NC	96.22%	10.73
WYPY-13	NC	79.76%	4.58
WYPY-14	NC	98.49%	16.87
WYPY-15[a]	NC	51.93%	2.85
WYPY-16	NC	97.38%	13.39
WYPY-17[a]	NC	64.24%	3.39
WYPY-18[a]	NC	46.02%	3.46
WYPY-19[a]	NC	40.28%	3.08
WYPY-20[a]	NC	48.43%	3.09
WYPY-21[a]	NC	54.73%	3.34
LMT-01	NC	99.19%	27.71
LMT-02[a]	NC	99.68%	81.78
LMT-03	NC	99.66%	60.07
LMT-04[a]	NC	28.23%	2.36
LMT-05	NC	96.31%	12.07
LMT-06[a]	NC	59.56%	3.05
LMT-07[a]	NC	94.90%	9.75
LMT-08	NC	98.17%	16.88
LMT-09[a]	NC	94.20%	9.05
LMT-10[a]	NC	75.64%	3.94
LMT-11[a]	NC	69.62%	3.57
LMT-12	NC	83.58%	5.09
LMT-13[a]	NC	40.90%	2.49
LMT-14[a]	NC	25.14%	2.14
LMT-15	NC	79.61%	4.39
LMT-16[a]	NC	23.48%	2.17
LMT-17[a]	NC	41.66%	2.51
LMT-18[a]	NC	21.64%	2.18
LMT-19[a]	NC	36.08%	2.29
LMT-20[a]	NC	27.29%	2.15
LMT-21	NC	92.77%	8.07
LMT-22[a]	NC	96.17%	11.74
LMT-23	NC	92.13%	7.59
GL-01[a]	NC	53.95%	3.71

GL-02	NC	96.99%	18.03
GL-03	NC	99.64%	57.17
GL-04[a]	NC	28.49%	2.36
GL-05[a]	NC	34.43%	2.57
GL-06[a]	NC	56.92%	3.06
GL-07[a]	NC	29.83%	2.45
GL-08[a]	NC	71.50%	4.25
GL-09	NC	99.19%	28.49
GL-10[a]	NC	99.08%	27.69
GL-11[a]	NC	97.40%	14.88
GL-12[a]	NC	96.00%	13.39
GL-13	NC	99.81%	78.25
GL-14	NC	99.47%	30.52
GL-15	NC	99.86%	98.08
GL-16[a]	NC	96.13%	10.72
GL-17[a]	NC	75.45%	4.31
GL-18[a]	NC	99.63%	39.19
GL-19[a]	NC	96.17%	10.25
GL-20[a]	NC	96.59%	11.88
GL-21[a]	NC	97.99%	16.80
GL-22	NC	98.84%	18.48
GL-23[a]	NC	97.55%	14.33
SB-01	C	99.33%	31.60
SB-02	C	96.93%	12.98
SB-03	C	99.76%	74.44
SB-04	C	97.76%	15.59
SB-05	C	91.39%	6.87
SB-06	C	92.89%	7.62
SB-07	C	91.22%	7.41
SB-08	C	85.04%	5.24
SB-09	C	89.63%	7.12
SB-10	C	98.27%	19.27
SB-11	C	89.31%	6.45
SB-12	C	84.14%	5.32

SB-13[a]	C	76.09%	4.51
SB-14	C	76.43%	5.41
SB-15	C	96.92%	12.84
SB-16	C	93.67%	8.73
SB-17	C	86.37%	5.89
SB-18	C	86.50%	6.45
SB-19[a]	C	47.29%	2.64
SB-20	C	95.15%	9.93
SB-21[a]	C	70.11%	3.64
SB-22	C	75.85%	4.75
SB-23	C	85.41%	5.62
SB-24	C	92.43%	8.49
SB-25	C	78.65%	4.42
SB-26[a]	C	91.12%	7.15
SB-27	C	98.94%	23.11
SB-28[a]	C	60.41%	3.06
SB-29	C	93.45%	8.40
SB-30	C	91.82%	7.54
SB-31[a]	C	93.69%	9.20
SB-32	C	86.85%	5.68
SB-33	C	76.99%	4.74
SB-34	C	92.46%	8.49
SB-35	C	88.08%	7.20
SB-36	C	88.22%	6.07
SB-37[a]	C	74.99%	4.14
SB-38[a]	C	76.80%	4.18
SB-39	C	84.99%	5.80
SB-40[a]	C	56.34%	3.36
SB-41	C	83.05%	5.30
SB-42	C	93.33%	8.40
SB-43	C	84.15%	5.14
SB-44	C	86.07%	5.59
SB-45	C	83.17%	5.02
SB-46	C	90.95%	7.13

SB-47	C	78.58%	4.47
SB-48	C	94.76%	10.03
SB-49	C	89.92%	6.73
SB-50[a]	C	69.91%	3.62
GMR-01[a]	C	57.72%	3.15
GMR-02	C	93.58%	8.71
GMR-03[a]	C	67.17%	3.90
GMR-04	C	97.75%	16.75
GMR-05	C	92.05%	8.03
GMR-06	C	98.16%	18.82
GMR-07	C	99.27%	36.00
GMR-08[a]	C	72.37%	4.24
GMR-09	C	99.80%	90.63
GMR-10	C	89.67%	7.06
GMR-11	C	94.39%	9.14
GMR-12[a]	C	97.66%	16.01
GMR-13[a]	C	94.72%	9.50
GMR-14	C	99.50%	41.44
GMR-15[a]	C	98.55%	19.70
GMR-16	C	98.76%	21.28
GMR-17	C	98.33%	18.47
GMR-18	C	98.45%	18.99
GMR-19	C	99.14%	27.37
GMR-20[a]	C	97.20%	13.47
GMR-21	C	98.37%	17.99
GMR-22	C	95.31%	10.36
GMR-23	C	99.54%	39.48
GMR-24	C	98.83%	22.05
GMR-25[a]	C	58.36%	3.07
GMR-26[a]	C	95.03%	9.52
GMR-27	C	96.90%	12.74
GMR-28	C	85.97%	5.74
GMR-29	C	98.63%	19.74
XGQ-01[a]	C	99.42%	58.41

XGQ-02	C	96.58%	12.54
XGQ-03	C	98.99%	25.94
XGQ-04	C	99.88%	152.98
XGQ-05	C	99.79%	87.23
XGQ-06	C	99.76%	74.98
XGQ-07	C	85.47%	5.58
XGQ-08	C	99.58%	47.31
XGQ-09	C	99.39%	41.75
XGQ-10	C	99.20%	27.36
XGQ-11	C	99.75%	74.09
XGQ-12	C	99.90%	167.87
XGQ-13	C	99.78%	88.52
XGQ-14	C	99.19%	29.62
XGQ-15	C	99.74%	70.02
XGQ-16	C	99.71%	66.85
XGQ-17	C	98.71%	22.22
XGQ-18[a]	C	99.87%	122.90
XGQ-19	C	99.82%	104.01
XGQ-20	C	94.98%	10.06
XGQ-21	C	98.92%	23.91
XGQ-22	C	97.25%	13.94
XGQ-23	C	99.07%	45.11
XGQ-24	C	98.87%	33.76
XGQ-25	C	95.83%	14.52
GHQ-01	C	98.98%	24.65
GHQ-02	C	87.43%	5.81
GHQ-03	C	98.28%	17.29
GHQ-04	C	83.54%	5.85
GHQ-05	C	89.14%	6.35
GHQ-06	C	99.76%	75.11
GHQ-07	C	99.38%	38.28
GHQ-08	C	99.65%	77.28
GHQ-09	C	98.21%	15.93
GHQ-10	C	99.32%	29.82

GHQ-11	C	98.50%	19.61
GHQ-12	C	99.56%	44.80
GHQ-13	C	87.38%	6.34
GHQ-14	C	92.19%	7.62
GHQ-15	C	99.07%	26.04
GHQ-16	C	96.66%	12.40
GHQ-17	C	98.00%	17.02
GHQ-18	C	99.59%	57.07
GHQ-19[a]	C	44.01%	2.89
SKD-01	C	98.93%	27.41
SKD-02	C	98.85%	23.27
SKD-03	C	99.35%	33.99
SKD-04	C	99.78%	107.25
SKD-05	C	97.55%	16.20
SKD-06	C	99.62%	68.64
SKD-07	C	99.84%	129.76
SKD-08	C	98.22%	22.00
SKD-09	C	84.65%	6.06
SKD-10	C	99.56%	37.27
SKD-11	C	92.61%	7.97
SKD-12	C	85.58%	5.56
SKD-13	C	99.10%	26.76
SKD-14	C	92.07%	7.37
SKD-15	C	98.58%	18.85
SKD-16	C	98.67%	19.43
SKD-17	C	94.98%	9.30
SKD-18	C	97.38%	13.13
SKD-19	C	98.82%	20.64
SKD-20	C	89.98%	6.86
SKD-21	C	82.50%	4.86
SKD-22[a]	C	76.68%	4.06
SKD-23	C	98.72%	18.13
SKD-24[a]	C	73.87%	3.82
SKD-25[a]	C	98.23%	16.22

SKD-26	C	99.69%	52.55
SKD-27	C	91.04%	6.68
SKD-28	C	88.60%	5.88
SKD-29[a]	C	95.89%	10.18
SKD-30	C	99.21%	26.91
SKD-31	C	97.60%	14.40
YZXL-01	C	98.63%	19.68
YZXL-02	C	98.87%	35.87
YZXL-03	C	94.39%	12.36
YZXL-04	C	97.97%	22.34
YZXL-05[a]	C	97.76%	25.66
YZXL-06	C	99.10%	30.45
YZXL-07	C	73.76%	7.54
YZXL-08	C	90.53%	9.50
YZXL-09[a]	C	25.79%	2.44
YZXL-10	C	85.94%	5.73
YZXL-11[a]	C	57.27%	4.35
YZXL-12	C	84.02%	5.58
YZXL-13[a]	C	96.17%	10.32
YZXL-14	C	99.02%	22.13
YZXL-15[a]	C	68.88%	4.74
YZXL-16[a]	C	98.26%	17.03
YZXL-17[a]	C	49.98%	3.05
YZXL-18	C	98.29%	16.88
YZXL-19	C	96.13%	10.90
YZXL-20[a]	C	99.38%	33.69
YZXL-21	C	98.54%	18.84
YZXL-22	C	99.54%	41.49
MY-01	C	99.82%	94.40
MY-02	C	99.90%	211.22
MY-03	C	99.74%	72.97
MY-04	C	99.73%	85.81
MY-05	C	99.62%	50.74
MY-06	C	99.72%	75.61

MY-07[a]	C	46.84%	2.49
MY-08	C	99.88%	159.28
MY-09	C	94.03%	11.00
MY-10	C	99.26%	34.41
MY-11	C	99.85%	137.98
MY-12	C	95.59%	10.98
MY-13[a]	C	81.44%	4.85
MY-14	C	97.75%	15.16
MY-15	C	92.14%	7.60
MY-16	C	91.48%	7.17
MY-17	C	99.66%	53.32
MY-18	C	99.48%	38.40
MY-19[a]	C	99.23%	26.70
MY-20[a]	C	98.50%	18.17
MY-21[a]	C	98.85%	23.17
MY-22[a]	C	99.21%	27.80
MY-23[a]	C	97.64%	14.33
MY-24	C	99.77%	74.27
MY-25[a]	C	94.07%	8.42
MY-26	C	99.70%	66.24
MY-27	C	98.61%	19.64
MY-28	C	92.49%	7.64
YA-01[a]	C	40.24%	2.61
YA -02	C	95.17%	10.07
YA -03	C	96.28%	11.21
YA -04	C	96.26%	11.30
YA -05	C	96.88%	13.26
YA -06	C	94.61%	10.68
YA -07	C	97.76%	15.79
YA-08[a]	C	57.57%	3.27
YA -09[a]	C	73.59%	4.01
YA -10[a]	C	44.67%	2.77
YA -11	C	85.00%	5.58
YA -12	C	86.31%	5.92

YA -13	C	99.38%	34.81
YA-14	C	83.93%	5.15
YA-15[a]	C	62.04%	3.58
YA-16[a]	C	66.95%	4.31
YA-17[a]	C	69.36%	3.57
YA-18[a]	C	97.03%	11.44
YA-19	C	97.38%	12.98
AY-01[a]	C	98.56%	16.99
AY-02	C	90.49%	6.84
AY-03	C	98.97%	23.05
AY-04[a]	C	94.48%	10.19
AY-05	C	89.67%	6.52
AY-06	C	89.35%	6.36
AY-07	C	88.77%	6.57
AY-08	C	90.06%	6.38
AY -09[a]	C	41.58%	2.51
AY -10	C	90.18%	6.44
AY -11	C	82.95%	4.95
AY -12	C	84.36%	5.46
AY -13[a]	C	71.26%	3.72
AY -14	C	93.07%	9.81
AY -15[a]	C	22.15%	2.07
AY -16	C	96.81%	12.98
AY -17[a]	C	61.91%	3.43
AY -18[a]	C	51.67%	2.99
AY-19[a]	C	68.15%	3.70
AY-20	C	80.28%	4.87
AY-21[a]	C	27.97%	2.37
AY-22[a]	C	88.86%	5.85
JSC-01[a]	SE	93.01%	11.70
JSC-02	SE	98.56%	18.28
JSC-03	SE	98.21%	19.68
JSC-04	SE	99.57%	38.79
JSC-05	SE	97.98%	18.53

JSC-06[a]	SE	27.95%	2.12
JSC-07	SE	98.35%	16.48
JSC-08[a]	SE	94.45%	8.72
JSC-09[a]	SE	75.92%	4.35
JSC-10[a]	SE	97.34%	15.05
JSC-11	SE	93.99%	8.36
JSC-12	SE	98.48%	17.84
JSC-13	SE	91.03%	7.17
JSC-14	SE	96.55%	14.30
JSC-15	SE	99.68%	47.00
JSC-16[a]	SE	48.39%	3.09
JSC-17[a]	SE	75.32%	4.23
JSC-18[a]	SE	64.89%	3.67
JSC-19[a]	SE	59.84%	3.28
JSC-20	SE	78.82%	4.63
JSC-21[a]	SE	62.34%	3.56
JSC-22	SE	98.40%	16.23
JSC-23	SE	97.69%	13.73
JSC-24[a]	SE	98.19%	15.30
JSC-25[a]	SE	64.60%	3.52
JSC-26[a]	SE	23.08%	2.53
JSC-27	SE	89.20%	6.39
JSC-28[a]	SE	47.41%	2.95
JSC-29[a]	SE	25.73%	2.46
JSC-30	SE	99.46%	35.55
JSC-31[a]	SE	86.23%	5.90
JSC-32[a]	SE	26.90%	2.38
CYS-01	SW	89.06%	7.50
CYS-02	SW	98.89%	27.10
CYS-03	SW	99.63%	64.11
CYS-04[a]	SW	90.96%	7.76
CYS-05	SW	99.62%	44.54
CYS-06[a]	SW	99.42%	30.91
CYS-07	SW	99.91%	204.51

CYS-08	SW	99.50%	43.58
CYS-09[a]	SW	99.09%	32.65
CYS-10	SW	99.79%	86.43
CYS-11[a]	SW	99.18%	33.51
CYS-12	SW	98.34%	22.20
CYS-13[a]	SW	72.79%	3.96
CYS-14	SW	91.66%	8.44
CYS-15	SW	99.69%	62.99
CYS-16[a]	SW	21.46%	2.22
CYS-17[a]	SW	30.44%	2.54
CYS-18[a]	SW	93.84%	10.11
CYS-19	SW	96.35%	11.79
CYS-20[a]	SW	97.64%	17.33
CYS-21[a]	SW	99.52%	39.80
CYS-22[a]	SW	47.46%	2.72
CYS-23	SW	84.47%	5.90
CYS-24[a]	SW	39.93%	2.68
CYS-25[a]	SW	45.41%	2.86
CYS-26[a]	SW	32.27%	2.31
CYS-27	SW	96.89%	14.34
CYS-28[a]	SW	98.75%	21.09
CYS-29[a]	SW	77.04%	4.32
CYS-30[a]	SW	99.45%	30.96
CYS-31[a]	SW	60.44%	3.42
LSS-01	SW	97.85%	18.50
LSS-02	SW	99.31%	30.22
LSS-03[a]	SW	61.91%	3.82
LSS-04	SW	99.10%	26.76
LSS-05[a]	SW	47.78%	2.85
LSS-06[a]	SW	41.28%	2.87
LSS-07	SW	98.38%	15.77
LSS-08[a]	SW	44.26%	2.74
LSS-09	SW	99.52%	30.90
LSS-10[a]	SW	57.53%	3.31

LSS-11	SW	96.37%	11.81
LSS-12	SW	99.52%	29.23
LSS-13[a]	SW	92.16%	10.74
LSS-14	SW	98.45%	18.78
LSS-15[a]	SW	73.02%	4.46
LSS-16	SW	98.13%	16.01
LSS-17[a]	SW	44.70%	2.89
LSS-18[a]	SW	86.59%	6.31
LSS-19[a]	SW	75.32%	4.30
LSS-20[a]	SW	41.51%	2.67
LSS-21[a]	SW	57.08%	3.12
LSS-22[a]	SW	56.16%	3.53
LMS-01	SW	98.94%	27.25
LMS-02[a]	SW	53.23%	3.26
LMS-03	SW	98.61%	21.15
LMS-04	SW	77.89%	9.23
LMS-05[a]	SW	68.82%	3.88
LMS-06	SW	98.95%	29.94
LMS-07[a]	SW	29.33%	2.27
LMS-08[a]	SW	41.44%	3.10
LMS-09	SW	94.04%	15.82
LMS-10	SW	98.86%	22.18
LMS-11[a]	SW	88.59%	12.05
LMS-12[a]	SW	98.27%	22.21
Rr3	*R. roxellana*	99.73%	28.33
Rr19	*R. roxellana*	99.24%	27.30
Rr21	*R. roxellana*	99.94%	27.23

Note: [a] Individual with mean depth < 4-fold or missing rate > 30% of genotypes across all target sites, or 1st-degree relatives.

Table S2. The eleven landscape variables and contributions considered in the resistance analysis.

Type	Variable	Data source	Percent contribution (%)
Natural environment	Altitude	https://www.nasa.gov/	1.3
	Slope	https://www.nasa.gov/	2.1
	**Aspect**	https://www.nasa.gov/	9.7
	Temperature	https://data.tpdc.ac.cn/home	4.2
	Precipitation	https://data.tpdc.ac.cn/home	2.9
	**Waterways**	https://www.openstreetmap.org	7.1
	**Vegetation**	https://www.resdc.cn	6.9
Human interference	**Roads**	(https://www.openstreetmap.org	10.1
	**Human settlements (Hs)**	(https://www.openstreetmap.org	30.7
	**Human population density (Hpop)**	https://www.worldpop.org	22
	Land use (LU)	https://www.webmap.cn/commres.do?method=globeIndex	3.1

Table S3. Bootstrapping results for *R. bieti* of 6 univariates with a contribution > 5% in MaxEnt (see Table S2).

No.	Model with landscape variable	Average AIC	Average weight	Average rank	Average LL	Percent top
1	Roads	240.63	0.13	9.55	-118.31	52.9
2	Aspect	245.80	0.02	28.38	-120.90	5.6
3	Vegetation	245.66	0.01	31.17	-120.83	0
4	Waterways	247.12	0.01	42.37	-121.56	4.3
5	Hs	247.65	0.00	45.43	-121.83	0
6	Hpop	248.93	0.01	48.15	-122.46	1.9

Note: The average AIC scores for the respective model are reported. The average weight, rank, and the mean log-likelihood of the parameter combination are also shown along with how many times the respective model was selected as the best model during 1,000 iterations.

Table S4. Bootstrapping results for *R. bieti* with 62 models including all possible combinations of covariates.

No.	Model with landscape variable combinations	Average AIC	Average weight	Average rank	Average LL	Percent top
1	Hs+Roads	242.66	0.03	10.95	-119.33	4
2	Roads+Vegetation	242.96	0.04	18.41	-119.48	2.7
3	Roads+Waterways	243.03	0.04	19.39	-119.51	0
4	Hpop+Roads	243.08	0.04	20.76	-119.54	0.1
5	Hpop+Roads+Waterways+Vegetation	243.30	0.02	14.74	-119.65	3.4
6	Aspect+Hpop+Vegetation	243.34	0.03	15.35	-119.67	4.9
7	Hs+Hpop+Roads+Waterways+Vegetation	243.38	0.02	15.49	-119.69	0.3
8	Aspect+Vegetation	243.38	0.03	15.77	-119.69	10.2
9	Aspect+Hpop+Roads+Vegetation	243.45	0.02	15.65	-119.72	0
10	Hs+Roads+Vegetation	243.81	0.02	18.66	-119.90	0
11	Hs+Vegetation	243.84	0.02	19.94	-119.92	0.9
12	Hpop+Roads+Waterways	244.14	0.03	26.86	-120.07	2
13	Aspect+Roads+Vegetation	244.14	0.01	22.23	-120.07	0
14	Aspect+Hs+Vegetation	244.42	0.02	24.25	-120.21	0
15	Hs+Roads+Waterways+Vegetation	244.43	0.01	31.14	-120.22	0
16	Aspect+Hpop+Roads+Waterways+Vegetation	244.44	0.01	29.78	-120.22	0
17	Aspect+Hs+Hpop+Roads+Vegetation+Waterways	244.61	0.01	30.99	-120.30	0
18	Aspect+Roads+Waterways+Vegetation	244.71	0.01	31.37	-120.36	0
19	Aspect+Hs+Waterways+Vegetation	244.72	0.01	30.01	-120.36	0
20	Aspect+Hs+Roads+Vegetation	244.75	0.02	26.06	-120.38	0
21	Hpop+Roads+Vegetation	244.82	0.02	25.50	-120.41	0

22	Roads+Waterways+Vegetation	244.83	0.01	29.09	-120.41	0
23	Hs+Hpop+Roads+Vegetation	244.85	0.01	30.03	-120.42	0.1
24	Aspect+Waterways+Vegetation	244.88	0.01	27.55	-120.44	0
25	Aspect+Hs+Roads+Waterways+Vegetation	244.90	0.01	34.04	-120.45	0
26	Hs+Waterways+Vegetation	244.95	0.01	32.18	-120.48	0
27	Aspect+Hpop	244.96	0.01	28.40	-120.48	0
28	Hpop+Waterways+Vegetation	244.99	0.01	31.63	-120.50	0
29	Aspect+Hpop+Waterways	245.02	0.01	29.98	-120.51	0
30	Aspect+Hs+Roads	245.02	0.01	29.48	-120.51	0
31	Aspect+Hs+Hpop+Waterways+Vegetation	245.04	0.01	36.63	-120.52	0
32	Hs+Hpop+Vegetation	245.04	0.02	29.92	-120.52	0.2
33	Aspect+Hs	245.09	0.01	25.66	-120.54	0
34	Aspect+Roads	245.10	0.01	25.63	-120.55	0
35	Aspect+Hpop+Roads	245.14	0.01	32.55	-120.57	0
36	Aspect+Hs+Hpop+Vegetation	245.18	0.01	34.38	-120.59	0
37	Aspect+Waterways	245.32	0.01	28.15	-120.66	0.5
38	Hpop+Vegetation	245.34	0.01	35.39	-120.67	0
39	Hs+Hpop+Waterways+Vegetation	245.51	0.01	33.81	-120.76	0.1
43	Aspect+Hpop+Waterways+Vegetation	246.27	0.01	35.62	-121.13	0
44	Aspect+Hs+Hpop+Roads+Vegetation	246.30	0.01	41.67	-121.15	0
45	Aspect+Hpop+Roads+Waterways	246.89	0.01	40.04	-121.44	2.6
46	Aspect+Roads+Waterways	246.95	0.01	41.78	-121.47	0
48	Hpop+Waterways	247.15	0.01	42.81	-121.58	0.1
49	Hs+Hpop+Waterways	247.19	0.01	43.46	-121.60	0
50	Hs+Hpop+Roads+Waterways	247.25	0.01	44.24	-121.63	0
51	Aspect+Hs+Hpop+Roads+Waterways	247.31	0.01	43.86	-121.65	1.4

52	Aspect+Hs+Roads+Waterways	247.55	0.01	45.86	-121.77	0.3
54	Aspect+Hs+Hpop+Waterways	247.69	0.01	46.63	-121.84	1.2
55	Hs+Roads+Waterways	248.17	0.00	45.72	-122.09	0
56	Aspect+Hs+Waterways	248.35	0.00	46.53	-122.18	0
57	Aspect+Hs+Hpop+Roads	248.53	0.00	48.35	-122.27	0.2
58	Hs+Waterways	248.57	0.00	48.02	-122.28	0
59	Hs+Hpop	248.57	0.00	48.09	-122.29	0
60	Hs+Hpop+Roads	248.61	0.00	49.04	-122.30	0
61	Aspect+Hs+Hpop	248.74	0.00	49.31	-122.37	0.1
62	Null	255.44	0.10	50.27	-125.72	18.30

Table S5. Demographic inferences of the *R. bieti* populations by Fastsimcoal2 and AIC values for models.

Models	AIC	Number of parameters
Model 1	6,047,560	17
Model 2	5,812,958	18
Model 3	5,812,131	18
**Model 4**	5,719,383	19
Model 5	6,036,734	14
Model 6	5,793,425	17

Note: The smallest AIC value indicated the best fit model.

Table S6. Area under the curve (AUC) values of ecological niche modeling across four periods: the Last Interglacial (LIG; ~129-116 ka), the Last Glacial Maximum (LGM; ~27-19 ka), the Mid-Holocene (MH; ~8.2–4.2 ka), and the present (1970-2000 AD).

**Periods**	**AUC**
LIG	0.772
LGM	0.775
MH	0.785
Present	0.821

Table S7. Distribution of targeted SNPs across each chromosome of the *R. bieti* genome.

Chromosomes	Number of SNPs
chr01	2,707
chr02	3,853
chr03	2,900
chr04	3,106
chr05	4,352
chr06	3,818
chr07	3,400
chr08	3,271
chr09	2,724
chr10	1,318
chr11	2,464
chr12	2,517
chr13	2,177
chr14	2,353
chr15	2,312
chr16	1,434
chr17	2,378
chr18	1,764
chr19	2,560
chr20	1,751
chr21	1,733
Total	54,892

Table S8. The nineteen bioclimatic variable and contributions considered in the ecological niche models.

Variable	Describe	Percent contribution (%)
bio1	Annual Mean Temperature	3
**bio2**	Mean Diurnal Range (Mean of monthly (max temp - min temp))	31
**bio3**	Isothermality	5.1
bio4	Temperature Seasonality	3.1
**bio5**	Max Temperature of Warmest Month	30.3
bio6	Min Temperature of Coldest Month	0.9
**bio7**	Temperature Annual Range (BIO5-BIO6)	7.3
bio8	Mean Temperature of Wettest Quarter	0.3
bio9	Mean Temperature of Driest Quarter	0.3
bio10	Mean Temperature of Warmest Quarter	0.8
bio11	Mean Temperature of Coldest Quarter	1.2
bio12	Annual Precipitation	5.5
bio13	Precipitation of Wettest Month	1.5
bio14	Precipitation of Driest Month	1.5
bio15	Precipitation Seasonality (Coefficient of Variation)	1.4
bio16	Precipitation of Wettest Quarter	0.9
bio17	Precipitation of Driest Quarter	3
bio18	Precipitation of Warmest Quarter	0.5
bio19	Precipitation of Coldest Quarter	2.3
